# Supplementary material for: Exploratory study of staff perceptions of shift safety in the critical care unit and routinely available data on workforce, patient and organisational factors
Source: BMJ Open. 2020 Jun 17;10(6):e034101. doi: 10.1136/bmjopen-2019-034101 (PMC7304836; doi:10.1136/bmjopen-2019-034101)

SUPPLEMENTARY MATERIALS

Supplementary File

Supplementary Table 1 Department of Health 2009 Levels of Care classification

Level 0	Patients whose needs can be met through normal ward care in an acute hospital
Level 1	Patients at risk of their condition deteriorating, or those recently relocated from higher levels of care, whose needs can be met on an acute ward with additional advice and support from the Critical Care team
Level 2	Patients requiring more detailed observation or intervention including support for a single failing organ system or post-operative care and those 'stepping down' from higher levels of care
Level 3	Patients requiring advanced respiratory support alone, or basic respiratory support together with support of at least two organ systems. This level includes all complex patients requiring support for multi-organ failure.

Supplementary Table 2 Characteristics of ICU sites during data collection

**Site**	**Number of available beds**	**Number of side rooms**	**Core Specialities**
Site 1	32	16	Major Trauma Centre, Vascular, General
Site 2	16	4	Heart Attack Centre, Hepatobiliary, Renal and transplant, Haematology/Oncology
Site 3	24	5	Stroke centre, Neurological, Neurosurgical, Head and Neck surgery, General.

Supplementary Table 3a Safer Nursing Care Tool

Level of Care	Descriptor
Level 0<br>Patient requires hospitalisation Needs met by provision of normal ward carers.	**Care requirements may include the following**<br>**• Elective medical or surgical admission**<br>**• May have underlying medical condition requiring on-going treatment**<br>**• Patients awaiting discharge**<br>**• Post-operative/post-procedure care - observations recorded half hourly initially then 4-hourly**<br>**• Regular observations 2 - 4 hourly**<br>**• Early Warning Score is within normal threshold.**<br>**• ECG monitoring**<br>**• Fluid management**<br>**• Oxygen therapy less than 35%**<br>**• Patient controlled analgesia**<br>**• Nerve block**<br>**• Single chest drain**<br>**• Confused patients not at risk**<br>**• Patient requires assistance with some activities of daily living, requires the assistance of one person to mobilise, or experiences occasional incontinence**
Level 1a<br>Acutely ill patients requiring intervention or those who are UNSTABLE with a GREATER POTENTIAL to deteriorate	**Care requirements may include the following**<br>**• Increased level of observations and therapeutic interventions**<br>**• Early Warning Score - trigger point reached and requiring escalation.**<br>**• Post-operative care following complex surgery**<br>**• Emergency admissions requiring immediate therapeutic intervention.**<br>**• Instability requiring continual observation/invasive monitoring**<br>**• Oxygen therapy greater than 35% +/- chest physiotherapy 2-6 hourly**<br>**• Arterial blood gas analysis - intermittent**<br>**• Post 24 hours following insertion of tracheostomy, central lines,epidural or multiple chest or extra ventricular drains**<br>**• Severe infection or sepsis**
Level 1b<br>Patients who are in a STABLE condition but are dependant on nursing care to meet most or all of the activities of daily living	**Care requirements may include the following**<br>**• Complex wound management requiring more than one nurseor takes more than one hour to complete.**<br>**• VAC therapy where ward-based nurses undertake the treatment**<br>**• Patients with Spinal Instability /Spinal Cord Injury**<br>**• Mobility or repositioning difficulties requiring the assistance of two people**<br>**• Complex Intravenous Drug Regimes - (including those requiring prolonged preparatory /administration/post-administration care)**<br>**• Patient and/or carers requiring enhanced psychological support owing to poor disease prognosis or clinical outcome**<br>**• Patients on End of Life Care Pathway**

	• **Confused patients who are at risk or requiring constant supervision**<br>• **Requires assistance with most or all activities of daily living**<br>• **Potential for self-harm and requires constant observation**<br>• **Facilitating a complex discharge where this is the responsibility of the ward-based nurse**
Level 2<br>May be managed within clearly identified, designated beds, resources with the required expertise and staffing level OR may require transfer to a dedicated Level 2 facility /unit	**Deteriorating/ compromised single organ system**<br>• **Post operative optimisation (pre-op invasive monitoring)/extended post-op care.**<br>• **Patients requiring non-invasive ventilation/respiratory support;**<br>**CPAP/BiPAP in acute respiratory failure**<br>• **First 24 hours following tracheostomy insertion**<br>• **Requires a range of therapeutic interventions including:**<br>• **Greater than 50% oxygen continuously**<br>• **Continuous cardiac monitoring and invasive pressure monitoring**<br>• **Drug Infusions requiring more intensive monitoring e.g. vasoactive drugs (amiodarone, inotropes, gtn) or potassium, magnesium**<br>• **Pain management - intrathecal analgesia**<br>• **CNS depression of airway and protective reflexes**<br>• **Invasive neurological monitoring**
Level 3<br>Patients needing advanced respiratory support and/or therapeutic support of multiple organs.	**Monitoring and supportive therapy for compromised/ collapse of two or more organ/ systems**<br>• **Respiratory or CNS depression/ compromise requires mechanical/invasive ventilation**<br>• **Invasive monitoring, vasoactive drugs, treatment of hypovolaemia/haemorrhage/ sepsis or neuro protection**

Supplementary Table 3b

SafeCare Allocate Colour	SafeCare Allocate Calculation	Category for analysis
Green	90% (underutilisation)	5
None given	90.1%-105%	4
Amber	105 % (overutilisation)	3
None Given	105.1%-110%	2
Red	>110% (overutilisation)	1

Supplementary Table 4 Summary of Responses

	Total		Site 1		Site 2		Site 3	
	Total	%	Total	%	Total	%	Total	%
Green	1731	61%	495	39%	685	83%	551	74%
Amber	523	18%	304	24%	101	12%	118	16%
Red	582	21%	461	37%	44	5%	77	10%
Total responses	2836		1260		830		746	
Response rate	57.7%		56.4%			70.8%		46.0%

Supplementary Table 5 Analysis of relationships between BPR and staffing, patient and work environment characteristics using mean, median and weighted mean BPR score.

Variable tested	BPR Score Mean*		BPR Score Median**		BPR Score Mean weighted ***	
	r value	p value	r value	p value	r value	p value
**CHPPD**	0.13	0.108	0.01	0.885	-0.11	0.151
**Safe care Allocate Score**	-0.19	0.013	-0.17	0.032	0.17	0.032
**% of level 1 patients**	-0.42	<0.0001	-0.34	<0.0001	0.41	<0.0001
**% of level 2 patients**	0.003	0.973	0.02	0.78	-0.0004	0.996
**% of level 3 patients**	0.32	<0.0001	0.22	0.004	-0.32	<0.0001
**Number of patients**	-0.6	<0.0001	-0.54	<0.0001	0.59	<0.0001
**% of patients in side rooms**	-0.45	<0.0001	-0.39	<0.0001	0.44	<0.0001
**% of staff with a CCC**	0.63	<0.0001	0.55	<0.0001	0.61	<0.0001

***each green response scores 3, each amber response scores 2, each red response scores 1, total score divided by the number of responses= BPR shift score. (3=safe 1 = very unsafe)**
****each green scores 3, each amber scores 2, each red scores 1, median score used= BPR shift score (3 =safe 1 =very unsafe)**
***** each green response scores 1, each amber scores 3, each green response scores 5, total score divided by the number of responses= BPR shift score (1= safe 5 = very unsafe)**

Supplementary Figure 1

Image 1

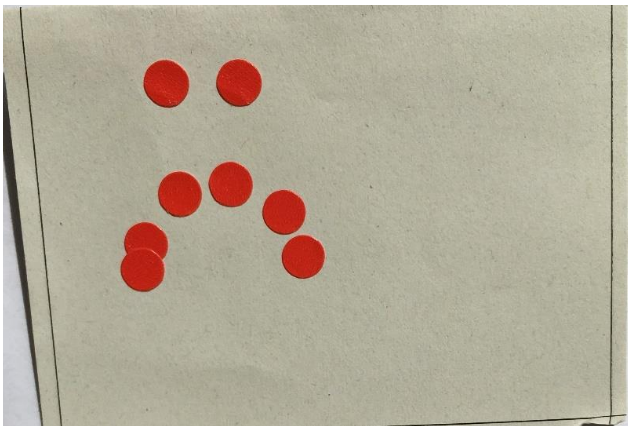

Supplement: Supplementary data [file bmjopen-2019-034101supp001.pdf]
