# Supplementary material for: Exploratory study of staff perceptions of shift safety in the critical care unit and routinely available data on workforce, patient and organisational factors
Source: BMJ Open. 2020 Jun 17;10(6):e034101. doi: 10.1136/bmjopen-2019-034101 (PMC7304836; doi:10.1136/bmjopen-2019-034101)
Supplement: Author's manuscript [file bmjopen-2019-034101.draft_revisions.pdf]

BMJ Open

BMJ Open is committed to open peer review. As part of this commitment we make the peer review history of every article we publish publicly available.

When an article is published we post the peer reviewers' comments and the authors' responses online. We also post the versions of the paper that were used during peer review. These are the versions that the peer review comments apply to.

The versions of the paper that follow are the versions that were submitted during the peer review process. They are not the versions of record or the final published versions. They should not be cited or distributed as the published version of this manuscript.

BMJ Open is an open access journal and the full, final, typeset and author-corrected version of record of the manuscript is available on our site with no access controls, subscription charges or pay-per-view fees (http://bmjopen.bmj.com).

If you have any questions on BMJ Open's open peer review process please email info.bmjopen@bmj.com

BMJ Open

# An exploratory study of staff perceptions of shift safety in the critical care unit and routinely available data on workforce, patient and organisational factors.

| | |
|---|---|
| Journal: | *BMJ Open* |
| Manuscript ID | bmjopen-2019-034101 |
| Article Type: | Original research |
| Date Submitted by the Author: | 05-Sep-2019 |
| Complete List of Authors: | Leon-Villapalos, Clare; Imperial College Healthcare NHS Trust, Critical Care<br>Wells, Mary; Imperial College London; Imperial College Healthcare NHS Trust, Directorate of Nursing<br>Brett, Stephen; Imperial College London, Surgery and Cancer; Imperial College Healthcare NHS Trust, Critical Care |
| Keywords: | Adult intensive & critical care < ANAESTHETICS, Safety, Staff deployment |
| | |

SCHOLARONE™
Manuscripts

*I, the Submitting Author has the right to grant and does grant on behalf of all authors of the Work (as defined in the below author licence), an exclusive licence and/or a non-exclusive licence for contributions from authors who are: i) UK Crown employees; ii) where BMJ has agreed a CC-BY licence shall apply, and/or iii) in accordance with the terms applicable for US Federal Government officers or employees acting as part of their official duties; on a worldwide, perpetual, irrevocable, royalty-free basis to BMJ Publishing Group Ltd ("BMJ") its licensees and where the relevant Journal is co-owned by BMJ to the co-owners of the Journal, to publish the Work in this journal and any other BMJ products and to exploit all rights, as set out in our licence.*

*The Submitting Author accepts and understands that any supply made under these terms is made by BMJ to the Submitting Author unless you are acting as an employee on behalf of your employer or a postgraduate student of an affiliated institution which is paying any applicable article publishing charge ("APC") for Open Access articles. Where the Submitting Author wishes to make the Work available on an Open Access basis (and intends to pay the relevant APC), the terms of reuse of such Open Access shall be governed by a Creative Commons licence – details of these licences and which Creative Commons licence will apply to this Work are set out in our licence referred to above.*

*Other than as permitted in any relevant BMJ Author's Self Archiving Policies, I confirm this Work has not been accepted for publication elsewhere, is not being considered for publication elsewhere and does not duplicate material already published. I confirm all authors consent to publication of this Work and authorise the granting of this licence.*

For peer review only

**TITLE PAGE**

**Title of the article: An exploratory study of staff perceptions of shift safety in the critical care unit and routinely available data on workforce, patient and organisational factors.**

Clare Leon-Villapalos [1], Mary Wells [2], Stephen J Brett [3]

[1] Corresponding author Clare Leon-Villapalos, Intensive Care Unit, Imperial College Healthcare NHS Trust. 9th Floor QEQM St. Mary's Hospital, Praed Street London W21NY clare.leon-villapalos@nhs.net Telephone 02033126666.

[2] Professor Mary Wells, Professor of Practice (Cancer Nursing), Imperial College London and Lead Nurse for Research, Directorate of Nursing, Imperial College Healthcare NHS Trust. London. UK.

[3] Professor Stephen J Brett. Professor of Critical Care, Dept. of Surgery and Cancer, Imperial College London, Consultant in Intensive Care Medicine, Imperial College Healthcare NHS Trust. London. UK.

Word count: 3956

Author ORCID ID

CLV 0000-0002-1610-0319

MW 0000-0001-5789-2773

SJB 0000-0003-4545-8413

**Keywords**

- Safety
- Critical care
- Staff deployment

For peer review only

## ABSTRACT

**Objectives**

To explore: bedside professional reported (BPR) perceptions of safety in intensive care staff; and the relationships between BPR safety, staffing, patient and work environment characteristics.

**Design** An exploratory study of self-recorded staff perceptions of shift safety and routinely collected data.

**Setting** A large teaching hospital comprising 70 critical care beds.

**Participants** All clinical staff working in adult critical care.

**Interventions** Staff recorded whether their shift felt "safe, unsafe or very unsafe" for 29 consecutive days. We explored these perceptions and relationships between these and routine data on staffing, patient and environmental characteristics.

**Outcome measures** Relationships between BPR safety and staffing, patient and work environment characteristics.

**Results** 2836 BPR scores were recorded over 29 consecutive days (response rate 57.7%). Perceptions of safety varied between staff, including within the same shift. There was no correlation between perceptions of safety and two measures of staffing; care hours per patient day (r= 0.13 p=0.108) and Safecare Allocate (r= -0.19 p=0.013). We found a significant, positive relationship between perceptions of safety and the percentage of Level 3 (most severely ill) patients (r=0.32, p=0.0001). There was a significant inverse relationship between perceptions of safety and the percentage of Level 1 patients on a shift (r= -0.42, p=<0.0001). Perceptions of safety correlated negatively with increased numbers of patients (r= -0.44, p=<0.0006) and higher percentage of patients located side rooms (r=0.63, p<0.0001). We found a significant relationship between perceptions of safety and the percentage of staff with a specialist critical care course (r=0.42. p=0.0001).

**Conclusion** Existing staffing models, which are primarily influenced by staff:patient ratios may not be sensitive to patient need. Other factors may be important drivers of staff perceptions of safety and should be explored further.

**Trial registration**- UK Health Research Authority approval was obtained (ID249248).


## STRENGTHS AND LIMITATIONS OF THE STUDY

- Our study included staff from all professions working in critical care.
- The study achieved a fair response rate from staff with complete information on patient status and organisational metrics.
- This exploratory study was from different intensive care units, but from the same overarching institution- thus we cannot be certain how far our observations can be generalised.
- In this study we were unable to explore the meaning behind why staff felt a particular shift was "safe" or not.

For peer review only

**FUNDING STATEMENT**

This work was supported by Imperial Health Charity, National Institute of Health Research (NIHR) Imperial Biomedical Research Centre funded predoctoral research grant (grant number RF18\100007).

**COMPETING INTERESTS**

The authors have no associations with commercial entities that provided support for the submitted manuscript or any financial or non-financial associations relevant to the submitted manuscript.

**INTRODUCTION**

Intensive care units (ICUs) are complex and expensive services where staffing is the greatest cost. Inadequate staffing can have serious consequences, (1, 2) and research suggests there is an interdependent relationship between the nature and composition of the workforce and patient outcomes, including mortality, in both acute, (3-8) and ICU (9-11) settings. The mechanisms that mediate these relationships are incompletely understood. (12) High quality, patient-centred care requires appropriately skilled and available personnel operating within staffing models that optimise their performance, but this may be difficult to achieve in the context of fiscal and labour market challenges. (13)

ICU specific staffing guidance, such as the United Kingdom Guidelines for the Provision of Intensive Care Services (14), is primarily based on staff:patient ratios, and the 'severity' of illness, as defined by the number of organs in failure, (15) (Supplementary table 1). The nature and intensity of critical care is, however, changing in response to multiple drivers including; technological advances, more numerous and more complex patients, an increased focus on survivorship and rehabilitation (16) and changing expectations of patients and families, therefore more complex models for staff deployment may be required.

Current approaches to safe staffing may not be sensitive to actual patient need or to rapid or unpredictable fluctuations in workload. Furthermore, it is unclear how tools to guide staffing establishments translate into staff deployment and staff *perceptions* of care. ICU workforce guidance (14) also tends towards a uni-professional "siloed" approach that does not reflect the current multi-professional nature of intensive care (17) or the significance of inter-professional dynamics for productivity. (18,19)

The degree to which existing models of staffing in ICU reflect issues of relevance to staff is also unclear, specifically, staff *perceptions* of "safety"- a term used colloquially to reflect an environment, human and physical, which minimises the risk of avoidable adverse events and deficits in care. Recent studies have indicated that perceptions of safety vary within institutions, (20) between individuals (21) and between staff and directors of institutions. (20) Perceived safety may also contribute to staff stress- particularly when there is a discrepancy between expectations and resources. (22, 23) Staff stress is recognised as prevalent and concerning (24) in the ICU workforce and may contribute to reduced quality of care, burnout and poor staff retention. (25)

Relationships between staff *perceptions* of "safety" and shift by shift variations in staffing metrics, staff deployment, patient and unit factors have not been explored in ICU. There have been few studies which examine the contribution and perspectives of all healthcare professionals within ICU, although this has been identified as a priority. (10,26).

For peer review only

This study therefore sought to contribute to our understanding of the complexity of staffing in ICU by:

Exploring staff perceptions of safety for all professions, on a shift by shift basis, using a bedside professional reported (BPR) safety estimate.

Exploring the relationships between BPR safety and routinely available data on; staffing, patient and work environment factors that influence deployment.

## METHODS.

This study was performed at Imperial College Healthcare NHS Trust, across 70 general critical care beds on 3 sites. Units vary in speciality focus, size and number of side rooms (Supplementary Table 2). UK Health Research Authority approval was obtained (ID249248). No patient or staff identifiable data were used, and explicit consent was not required. Data were collected over 29 consecutive days during October and November 2018. During the study period each site was visited or contacted daily to collect and verify data.

### Patient and Public Involvement

There were no funds or time allocated for patient or public involvement.

The target population, staff from all professional groups, were involved in informal discussion of the research question, directly participating in the study and in 'sense checking' the methods of analysis. Results of the study were disseminated to several staff forums, including those for people in leadership positions who direct staffing deployment.

### Data collection and measures

#### Bedside Professional Rating of Safety (BPR).

We asked all ICU staff to rate each shift as "safe, unsafe, or very unsafe" using a coloured sticker (green, amber or red). This question was generated from previous work into staff experiences of safety. (21) During staff briefings and daily visits to sites, participants were encouraged to interpret the term "safe" as their own perception of safe in its normal conversational sense in ICU.

Staff were asked to choose one sticker to rate each shift and put this onto a card labelled with the relevant date and shift (e.g. October 4$^{th}$ Night shift). Participants posted the cards in data collection boxes, allowing them to complete the process anonymously.

To allow us to look for relationships between perceptions of safety and other variables, including staffing, patient and work environment factors, a BPR shift score was created for each shift using the following method; we counted the responses and allocated a score of 1 for each red response, 2 for each amber response and 3 for each green response. We summed the total score and divided this by the number of responses for that shift. This created a mean shift safety score *between* 1 (very unsafe) and 3 (safe), which reflected the diversity and weighting of responses and is simple to reproduce. This method was discussed with senior nursing staff to confirm that they felt it reasonably summarized perceptions of safety on a given shift.

For peer review only

## Routinely available data on staffing, patient and work environment

**Staffing and workload data.**

We recorded the number of staff working clinically in the ICU during the study period. Staffing data were extracted from an electronic rostering system and verified with the nurse in charge. Where professional groups did not use electronic rostering e.g. physiotherapists, the data were collected daily and confirmed weekly with the relevant manager. Staff who worked clinically for part of a 12-hour shift were represented as a proportion of a whole-time equivalent (e.g. a physiotherapist working for 6 hours on ICU was counted as 0.5).

We calculated workload intensity scores by summing the total number of organs in failure for each day and dividing this by the number of staff working that day. We looked at this by profession and by all staff combined. We used data from the critical care minimum data set (CCMDS) (27) to calculate the number of organs each patient had in failure, this was only available by 24-hour period, and therefore, data were collapsed into a 24-hour period.

We recorded the care hours per patient day (CHPPD) from our electronic roster. CHPPD is a measure that can be used to assess productivity and facilitates benchmarking. (28) We recorded staffing utilization data from SafeCare in the commercial rostering system provided by Allocate (AllocateSoftware Richmond 2017). This uses the composite acuity/dependency scoring system from the Safer Nursing Care Tool (29) (Supplementary Table 3a) to provide nursing utilisation reports to support real-time deployment decisions. This is done by calculating required nursing hours (based on acuity and dependency data entered by senior nursing staff) and dividing this figure by the number of rostered nursing hours to produce a percentage of nursing hours utilization (e.g. required hours 126.4, rostered hours 115.00 = 109.9 % utilisation).

This figure is reported in the following (partially) colour coded categories: <90% Green, 90.1% -104.9%, 105% Amber, 105.1%- 109.9 %, 110.0% Red. There is meaning attached to these five categories, so that green is reported as underutilisation whilst all figures falling into amber and above are reported as overutilisation. In our analysis we reflected these categories by allocating a number to each so that green = 5 through to red =1. This is illustrated in detail in (Supplementary Table 3b).

During the study if we found within-shift changes in staff numbers, patient numbers or patient acuity we recorded the status at approximately 5:00 and 17:00 (day shifts). This was a pragmatic decision that reflected the timings of local processes to manage staffing.

**Patient characteristics data.**

Data regarding the number of Level 1,2 and 3 patients (15) were recorded by visiting or contacting each site daily. In ICU, patients are broadly categorised by the number of organs in failure, with Level 3 patients being the most severely ill (Supplementary table 1).

**Work environment data.**

We recorded the total number of patients and the number of patients in side rooms per shift and confirmed this daily with a senior nurse.

**Staff characteristics data.**

We recorded the number of nursing staff on each shift who had completed a post registration critical care course (CCC), the accuracy of the data were confirmed with local clinical nurse educators.

For peer review only

**Analysis**

Data were collated into an Excel spreadsheet (Microsoft, Redmond, Washington State, USA). We used descriptive statistics to analyse frequency of responses. Data were tested for normality using the D'Agostino-Pearson Omnibus test and non-parametric tests were used where appropriate. We used Spearman's rank correlation to evaluate strength and direction of relationships; regression lines and 95% confidence intervals were plotted for illustrative purposes. Prism (v8.01 for Windows, GraphPad Software, La Jolla California USA) was used throughout.

Although the shift BPR was primarily analysed as a mean for simplicity – we further explored this using both median and a weighted approach (whereby a weighting of 5 was given to very unsafe, 3 to unsafe and 1 to safe). The purpose of this was to allow us to explore the sensitivity of the BPR tool.

## RESULTS

**Exploring BPR perceptions of safety**

A total of 2836 BPR responses were received during 29 days of data collection at 3 sites, we received responses from 170 out of a total of 174 twelve-hour shifts (98% of shifts). The overall response rate, using a denominator of all ICU staff (that is all staff working on ICU, including those who were transient or only partially based on ICU) was 57.7 %. The combined responses are illustrated in figure 1a. Most responses were green "safe" (61%), 18 % of responses were amber, "unsafe" and 21% of responses were red, "very unsafe". Total responses and responses by site are illustrated in Supplementary Table 4.

We noted that staff perceptions frequently varied within the same shift; for only 13% of shifts did all staff who responded report the same perception of safety for their site.

We calculated a BPR shift safety rating, using the methods described previously, which summarised general shift responses and allowed us to look for relationships between reported shift safety and staffing, patient and work environment characteristics. The range of mean shift BPR scores was 1.00 - 3.00, median 2.63. The distribution of the BPR shift scores for 170 shifts is illustrated in Figure 1b.

The BPR tool summarizes the overall feeling of a shift by incorporating all responses but does not differentiate between shifts with low and high response rates. To address this we looked to see if there was a relationship between response rates and perceptions of safety which might reflect a systematic bias, we found a weak relationship between staff perceiving a shift to be safe and a higher response rate ($r = 0.156$, $p= 0.0426$ Figure 1 c).

**Perceptions of safety and staffing data.**

We did not find a significant relationship between perceptions of safety and CHPPD ($r= 0.13$ $p=0.1080$, Figure 2a). We found a weak inverse relationship between perceptions of safety and staffing utilization (SafeCare Allocate) ($r = -0.19$, $p = 0.013$ Figure 2b).

We found a very weak relationship between perceptions of safety and workload "intensity" (defined as number of organs in failure/total number of nurses on shift). Looking only at nursing hours, staff reported feeling safer at higher rather than lower intensity workloads $r= 0.15$ $p=0.047$). We did not find a significant relationship between these variables when we included doctors' hours ($r =0.14$ $p=0.73$) and allied health professionals (AHP) hours ($r = 0.13$, $p=0.09$).

**Perceptions of safety and patient characteristics**

We found a significant, inverse relationship between perceptions of safety and the percentage of Level 1 patients on a shift (p=<0.0001, r= -0.42 Figure 3a). There was no significant relationship between perceptions of safety and the percentage of Level 2 patients (p=0.9729, r= 0.003 Figure 3b). We found a significant, positive, relationship between perceptions of safety and the percentage of Level 3 patients (p=0.0001, r= 0.32 Figure 3c).

**Perceptions of safety and the work environment**

We found a significant inverse relationship between perceptions of safety and numbers of patients in the largest unit (Unit 1) where number of patients ranged between 27 and 32 (figure 4a r=-0.44, p=<0.0006). We did not find a significant relationship in the smaller units (where numbers of patients were between 11-16 and 17-23 respectively figures 4b & c). We also found a significant inverse relationship between perceptions of shift safety and the percentage of patients who were occupying side rooms in a given shift (r= -0.45 P<0.0001 Figure 4d).
We found a significant, positive relationship between the percentage of nursing staff who had a critical care course on each shift and perceptions of safety (r = 0.63, p= <0.0001 Figure 5)

These analyses were repeated using both median scores and a score that gave additional weighting to "unsafe" responses, we found only minor differences in the relationships using these methodologies. These are summarised in Supplementary table 5.

**DISCUSSION**
Our study sought to explore an estimate of staff *perceptions* of shift safety for all professions in ICU on a shift by shift basis; we termed this bedside professional reported (BPR) safety. We also aimed to explore the relationships between this estimate and other factors, including routinely available data on staffing, patient and work environment factors that influence deployment. Staff perceptions of safety are important as they may reflect safety culture (30) and be associated with the risk of adverse events (31) including risks not identified by organisational processes. (1) We observed that staff found the BPR tool easy to implement and seemed acceptable to use (although no formal assessment of acceptability was made); previous pilots using more ambitious methods had proved unsuccessful due to poor engagement. The BPR's simplicity and fair completion rates are strengths and are important features of safety tools (30); it also yields unit level data, which could be used to inform local discussion and action. We are not aware of any studies of similar tools in ICU, and although further validation of the BPR tool is needed, the relationships we found were plausible. The very weak relationship we found between higher response rates and perceived safety indicates that we cannot rule out a systematic bias, with higher response rates on "safer" shifts. However, neither the strength or direction of the relationship is pronounced. Exploring the data using a median -based or weighted approach to BPR did not alter the observed relationships (Supplementary Table 5). Organisations wishing to create a more safety sensitive system might consider further adjustments to weighting.

For most shifts during the study period we found heterogeneity in reported perceptions of safety. Similarly, different "risk horizons" between and within staff groups on ICU, depending on team and environmental factors, have been previously described. (21) Both studies support an argument that

For peer review only - http://bmjopen.bmj.com/site/about/guidelines.xhtml

training for those with leadership roles in ICU should explicitly address heterogeneity of experience, perceptions of safety and anxiety amongst ICU staff.

Staff recorded their *perceptions* of shift safety; being involved in an enterprise which is not felt by the individual to be "safe" may be a source of stress. This was reflected in the way some staff responses seemed to be framed (which included annotations and pictures, Supplementary Image 1). We found during data collection, that staff described the stickers as "cathartic" suggesting that the BPR facilitated the articulation of feelings that were not captured elsewhere. It is reasonable to suggest that being able to communicate concerns about safety to someone who will listen may be important for staff wellbeing. This may be of particular importance in ICU, an environment where staff stress and burnout are areas of concern. (24,32)

Perceptions of shift safety were not well correlated with some nursing staffing metrics (NB deployment metrics for AHPs and medical staff are not currently in use at our organisation). The lack of relationship between these organisational measures and staff perceptions reinforces the importance of triangulating staff deployment decisions with professional judgment and suggests organisations should be cautious in relying solely on mathematically derived models. This may be one of the reasons that CHPPD is not consistently accepted by Nurse Directors (33) and it is already noted that SafeCareAllocate has not been validated for use in deployment. (34) Although the use of staffing benchmarks to prevent the erosion of staffing numbers is likely to remain important, the findings of this study suggest that more sophisticated thinking is needed to inform staffing models.

Our study found a significant relationship between patient acuity and perceptions of safety. Perhaps counterintuitively, more staff reported feeling safe when there were larger numbers of severely unwell (Level 3) patients, and reported feeling shifts to be "unsafe" when there were larger numbers of low acuity patients (Level 1). We did not specifically explore what mediated this relationship, but possible explanations include: that existing ICU staff deployment models are more sensitive to need for high acuity than for lower acuity patients; that staff are less confident in caring for this patient group; or that they are a marker for other workflow stressors, such as throughput or a very dynamic workload. This finding may indicate that uni-professional approaches that allocate fewer nursing hours to less severely unwell patients (where severity of illness based on categories of organ failure) may not match staff perceptions of patient need.

We found that size of unit and an increased proportion of patients located in side rooms were significantly related to staff reporting the shift as feeling less safe. We did not explore what mediated these relationships; possible explanations might include the impact of larger volumes of patients on individuals' capacity so that there may be a degree of activity which is beyond most staff's comfort zone. In the largest unit, processes were starting to be put in place to organise this as two smaller units but these were not fully embedded during the study period. Side rooms are increasingly common in ICU due to the benefits of improved patient privacy, confidentiality and infection control. However, there is little research into the impact on staff. Recent studies conducted in acute wards suggest that trade-offs for nursing staff include increased staff anxiety due to decreased surveillance and fewer opportunities for informal education, role modelling and interaction. (35,36) Our findings suggest that there may be value in exploring the relationships between staff perceptions of safety and unit design to identify modifiable factors, as larger units and side rooms become increasingly common features of ICU's.

For peer review only

The strong positive relationship between perceptions of safety and the percentage of staff on each shift with a CCC supports guidance that this should be a benchmarked target (14) and suggests that there may be value in training local establishments to a level which enables this to be achieved in daily staffing rotas. Our finding is consistent with the evidence that increased nursing education (to Bachelor level) is associated with a reduced likelihood of patients dying (4). It is also supported by recent findings (37), which indicated that strain and burnout in ICU clinicians is not inevitable and can be modified by an intensive education program**.**

## LIMITATIONS

Our study had several limitations:

Whilst the study included three separate sites, it was conducted at a single NHS Trust. Findings therefore may reflect factors unique to our culture and organisation, however the volume of observations and range of specialities suggest that our data may have broader applicability.

This was an exploratory study and the first use of the BPR scale, which although not formally "validated" seemed to have face validity with participants and senior staff. The intensity-derived data might not be sensitive to variations within a given shift or between sites. Furthermore, although it seems intuitively obvious, we cannot know for certain that working on a shift experienced as "unsafe" causes anxiety. We did not attempt to capture anxiety because in a preliminary study we found that attempting to acquire too much data at the end of a busy shift produced poor survey return and we were keen to avoid undermining the primary objective.

The question "How safe was your shift" was taken from associated work undertaken previously on our unit. The initial unpublished pilot was perceived as too complex and did not gain traction; we therefore reduced the original 5-point response options to "safe, unsafe and very unsafe". During the data collection we noted that staff often annotated their responses with extra stickers, drawings or comments, indicating that three responses did not always allow them to express fully the depth or range of feelings- which seemed more important at the "unsafe" end. Equally it could be argued that there is no meaningful difference between unsafe and very unsafe and that these categories could be collapsed, we therefore felt the option we selected was reasonable.

Early conversations with staff indicated that many staff did not want to indicate their professional group when reporting their perception of shift safety, as they felt this might compromise anonymity, we therefore did not ask for this information. This meant we were not able to analyse responses by professional group. Whilst this information would have brought valuable insights, it may have impacted on the response rates. Subsequent informal discussions have suggested that, having gained confidence in the tool and motivation behind the research project, staff would be willing to indicate their profession in future studies.

Despite a satisfactory response rate, we did not receive responses from all staff and therefore do not know whether the perceptions of staff who did not engage are different to those who did.

Most significantly, we do not know what lay behind different individual feelings of safety- or what might mediate the relationships we identified between perceptions of safety and the characteristics discussed. Further qualitative work is currently being undertaken to address this.

**IMPLICATIONS**

Understanding how to deploy the multi-professional team in a way that sensitively matches patient need in ICU is challenging and there is limited evidence available to inform guidance. Incorporating staff perceptions may be useful organisationally and may help identify modifiable factors that impact on staff stress and burnout.

The findings of our exploratory study suggest that

- A BPR tool is simple to use and seems acceptable to staff; it may capture near real-time staff perceptions of safety.
- Perceptions of safety vary between individuals on the same shift and this should be factored into the way teams are trained and led.
- Perceptions of safety may not align to some organisational staffing metrics, reinforcing the need to augment these with professional judgement and other measures.
- Further research could usefully focus on whether workforce deployment based on ratios and acuity is sensitive to perceived need, particularly for less acute patients in ICU.
- The composition of the workforce is important to staff perceptions of safety and our findings support the provision of specialist post-registration critical care course training for nursing staff.
- Further research could usefully focus on staff deployment for patients classified as less severely unwell and the impact of the size and layout of ICUs.

To our knowledge this is the first study to include allied health professionals in addition to nurses and doctors to explore perceptions of safety and staff deployment in ICU. This approach may yield results that better reflect the reality of how care is delivered in ICU and can also accommodate emerging roles such as nursing associates and advanced critical care practitioners. This study has relevance nationally; the factors explored reflect, or are similar to, guidance followed by most UK ICUs. The findings may be of interest outside of the UK since, whilst processes vary, there are shared concerns regarding workforce deployment, supply, costs and burnout.

For peer review only

**Legends for figures**

**Figure 1a** Bedside professional reported (BPR) safety October 4th-November 2nd (n=2836).

**Figure 1b** Distribution of bedside professional reported (BPR) safety - shift scores (n=170/ 174 shifts).

**Figure 1 c** Relationship between bedside professional reported (BPR) safety and response rates (n=170/ 174 shifts).

**Figure 2a** Relationship between bedside professional reported (BPR) shift safety and care hours per patient day (CHPPD) (n=170/ 174 shifts)

**Figure 2b** Relationship between bedside professional reported (BPR) shift safety (BPR) and safe staffing "score" 1=staff overutilization red, 5 = green staff underutilization (n=170/ 174 shifts)

**Figure 3 a, b & c** Relationship between bedside professional reported (BPR) shift safety and volumes of acute and less acute patients Level 1, Level 2, Level 3 (n=170/ 174 shifts)

**Figure 4 a, b & c** Relationships between bedside professional reported (BPR) shift safety and number of patients in unit 1 (n=58), 2(n=56) and 3(n=56), (n=58)

**Figure 4d** Relationship between bedside professional reported perceptions of shift safety (BPR) and the percentage of patients in a side room (n=170/ 174 shifts).

**Figure 5** Relationship between bedside professional reported (BPR) shift safety and the percentage of staff on a shift with a critical care course (n=170/ 174 shifts).

**All contributors were involved in:**

- Substantial contributions to the conception, design analysis, and interpretation of data for the work.
- Drafting the work or revising it critically for important intellectual content;
- Final approval of the version to be published;
- Agreement to be accountable for all aspects of the work in ensuring that questions related to the accuracy or integrity of any part of the work are appropriately investigated and resolve

For peer review only

**Acknowledgements**

**Funding Statement**

This work was supported by Imperial Health Charity, National Institute of Health Research (NIHR) Imperial Biomedical Research Centre funded predoctoral research grant (grant number RF18\100007).

**Competing Interests**

The authors have no associations with commercial entities that provided support for the submitted manuscript or any financial or non-financial associations relevant to the submitted manuscript.

**Data sharing statement**

All all available data will be submitted to the DRYAD repository

**REFERENCES**

1 Francis R. The Mid Staffordshire NHS Foundation Trust Public Inquiry. Report of the Mid Staffordshire NHS Foundation Trust Public Inquiry: Executive Summary. London: Stationery Office, 2013.

2 Berwick D.A promise to learn- a commitment to act: improving the safety if patients in England. (2013) London: Department of Health.

3 Griffiths P, Maruotti A, Recio Saucedo A, *et al.* Nurse staffing, nursing assistants and hospital mortality: retrospective longitudinal cohort study. *BMJ Qual Saf*;**28:**609-617. doi:10.1136/bmjqs-2018-008043.

4 Aiken LH, Sloane DM, Bruyneel L *et al*. Nurse Staffing and education and hospital mortality in nine European countries: a retrospective observational study. *The Lancet* 2014;**383**:1824-30. doi:10.1016/S0140-673(13)62631-8.

5 Aiken LH, Sloane D, Griffiths P, *et al*. Nursing skill mix in European hospitals: cross-sectional study of the association with mortality, patient ratings, and quality of care. *BMJ Qual Safety*2016*;***26**: 559-568doi: 10.1136/bmjqs-2016-005567.

6. Griffiths P, Ball J, Murrells T, *et al*. Registered nurse, healthcare support worker, medical staffing levels and mortality in English hospital trusts: a cross-sectional study. *BMJ Open* 2016;**6**:e008751.doi10.1136/bmjopen-2015-008751.

7 Needleman J, Buerhaus P, Pankratz VS, *et al.* Nurse staffing and inpatient hospital mortality. *N Engl J Med*2011;**364:**1037-45. doi:10.1056/NEJMsa1001025.

8 Rafferty AM, Clarke S, Coles *J et al*. Outcomes of variation in hospital nurse staffing in English hospitals: Cross-sectional analysis of survey data and discharge records. *Int J Nurs Stud*2007;**44** (2):175-182 doi: 10.1016/j.ijnurstu.2006.08.003.

For peer review only

9 Provonost PJ, Dang D, Dorman T *et al* Intensive care unit staffing and the risk for complications after abdominal aortic surgery. *Eff Clin Pract*2001;**4**:199-206.

10 West E, Baron DN, Harrison D *et al*. Nurse staffing, medical staffing and mortality in intensive care: an observational study. *Int J Nurs Stud*2014; **51:**781-94. doi:10.1016/j.ijnurstu.2014.02.00.

11 Hugonnet S, Uckay I & Pitter D. Staffing level: a determinant of late-onset ventilator-associated pneumonia. *Crit Care*2007;11 doi 10.1186/cc5974.

12 Leary A, Punshon G. Determining acute nurse staffing: a hermeneutic review of an evolving science. *BMJ Open*2019; **9**:e025654 doi 10.1136/bmjopen-2018-025654.

13 Borneo A. Staffing for Safe and Effective Care Nursing on the Brink. RCN Policy Report. 2018. London.

14 The Faculty of Intensive Care Medicine. Guidelines for the Provision of Intensive Care Services version 2. 2019 https://www.ficm.ac.uk/standards-research-revalidation/guidelines-provision-intensive-care-services-v2 accessed June 2019.

15 Department of Health. Comprehensive Critical Care: A Review of Adult Critical Care Services. 2000.

16 National Institute for Healthcare Excellence. Rehabilitation after critical illness in adults. Clinical guideline [CG83]. March 2009.

17 Donovan A Matthew Aldrich J Kendall Gross et al. Interprofessional care and teamwork in the ICU *Crit Care Med*2014;46**(6)**:1 doi 10.1097/CCM.0000000000003067.

18 Lalfond S, Charlesworth. A Year of plenty? (2017) The Health Foundation. London.

19 Hewitt C, Lankshear A, Kazanjn A *et al.* Health Service Workforce and Health Outcomes: A Scoping Study. Report for the National Co-ordinating Centre for NHS Service Delivery and Organisation. https://www.journalslibrary.nihr.ac.uk/programmes/hsdr/081319050/#/documentation accessed May 2019

20 Huang D, Clermont G, Sexton K *et al.* Perceptions of safety culture vary across the intensive care units of a single institution. *Crit Care Med*2007; 35**1:**165-176 doi10.1097/01.CCM.0000251505.76026.CF.

21 D'Lima D, Murray E, Brett S. Perceptions of Risk and Safety in the ICU: A Qualitative Study of Cognitive Processes Relating to Staffing *Crit Care Med*2018*;*46**1**:60-70. doi10.1097/CCM.0000000000002773.

22 Wolf L, Perhats C, Delao A *et al*. On the Threshold of Safety: A Qualitative Exploration of Nurses' Perceptions of Factors Involved in Safe Staffing Levels in Emergency Departments. *Journal of Emergency Nursing*2017;43**2:**150-157 doi 10.1016/j.jen.2016.09.003.

23 Vandevala T, Pavey L, Chelidoni O *et al.* Psychological rumination and recovery from work in intensive care professionals: Associations with stress, burnout, depression and health. *Journal of Intensive Care*2017;5**16** doi 10.1186/s40560-017-0209-0.

24 Seaman J, Cohen T, White. D. Reducing the stress on clinicians working in the ICU *JAMA* 2019; 320**19**:1981-1982 doi10.1001/jama.2018.14285.

For peer review only

25 Halter M, Boiko O, Pelone f *et al.* The determinants and consequences of adult nursing staff turnover a systematic review of systematic reviews *Health Services Research.* 17**1**:824 doi 10.1186/s12913-017-2707-0.

26 National Institute for Health Research Themed Review. Staffing on Wards. Making decisions about healthcare staffing improving effectiveness and supporting staff to care well. *NIHR Dissemination Centre* 2019 doi 10.3310/themedreview-03553La

27 Critical Care Minimum Dataset. Available from: http://www.datadictionary.nhs.uk/data_dictionary/messages/supporting_data_sets/data_sets/critic al_care_minimu m_data_set_fr.asp (accessed December2018)

28 NHS Improvements *Care hours per patient day (CHPPD): guidance for acute and acute specialist trusts (*2018) NHS Improvements https://improvement.nhs.uk/documents/3177/CHPPD_guidance_-_acute.pdf (accessed April 2019).

29 The Shelford Group. Safer nursing care tool implementation resource pack. The Shelford Group 2014.

30 The Health Foundation Measuring safety culture. The Health Foundation 2011.

31 Olds D, Aiken L, Cimiotti J *et al.* Association of nurse work environment and safety climate on patient mortality: A cross-sectional study. *Int J Nurs Stud*2017;**(74):**155-161 doi 10.1016/j.ijnurstu.2017.06.004

32 Wong A, Olusanya O. Burnout and resilience in anaesthesia and intensive care medicine. *BJA Education*2017;17**10:** 334-340 doi 10.1093/bjaed/mkx020

33 Ball J, Barker H, Griffiths P*et al.* Implementation, Impact and Costs of Policies for Safe Staffing in Acute NHS Trusts 2019 doi:10.5258/SOTON/P0012

34 Saville C, Griffiths P, Ball J *et al.* How many nurses do we need? A review and discussion of operational research techniques applied to nurse staffing. *Int J Nurs Stud*2019; **97:**7-13. doi10.1016/j.ijnurstu.2019.04.015

35 Maben J, Griffiths P, Penfold C *et al*. One size fits all? Mixed methods evaluation of the impact of 100% single-room accommodation on staff and patient experience, safety and costs *BMJ Qual Saf* 2016;**2**:241-256 doi:10.1136/bmjqs-2015-004265

36 Maben J, Griffiths P, Penfold C, *et al*. Evaluating a major innovation in hospital design: workforce implications and impact on patient and staff experiences of all single room hospital accommodation. *Health Serv Deliv Res*.2015;**3**:304. doi 10.3310/hsdr03030

37 El Khamali R, Mouaci A, Valera S, *et al;* for the SISTRESSREA Study Group. Effects of a multimodal program including simulation on job strain among nurses working in intensive care units: a randomized clinical trial. *JAMA2018;*.**320**:1988-1997doi:10.1001/jama.2018.14284

For peer review only

For peer review only

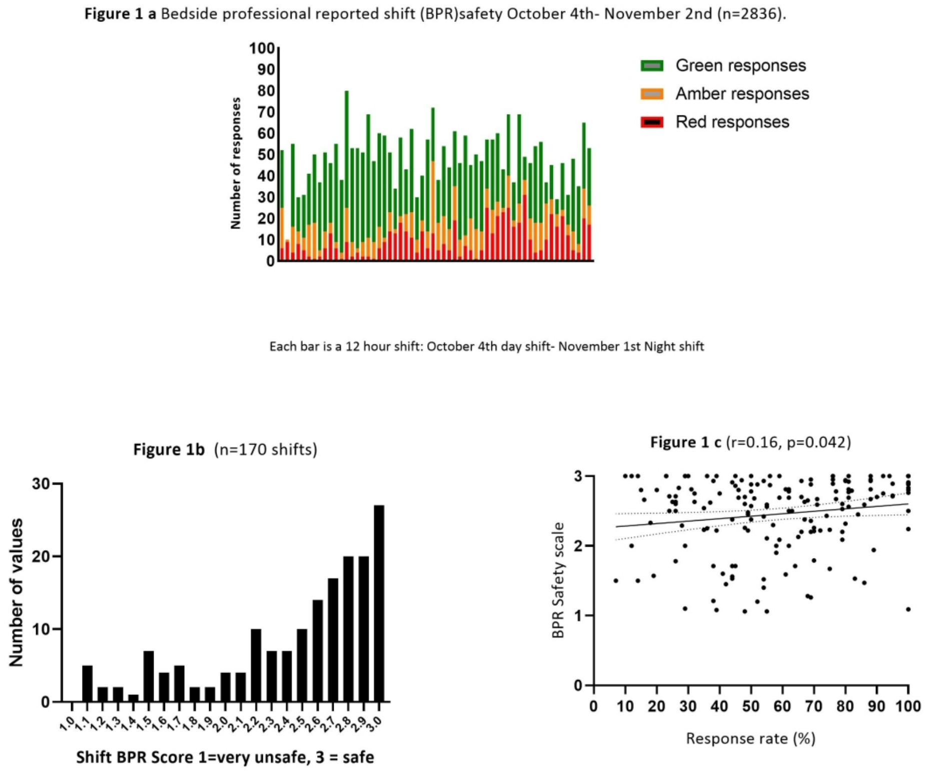


Figure 1a Bedside professional reported (BPR) safety October 4th-November 2nd (n=2836).
Figure 1b Distribution of bedside professional reported (BPR) safety - shift scores (n=170/ 174 shifts).
Figure 1 c Relationship between bedside professional reported (BPR) safety and response rates (n=170/ 174 shifts).

215x178mm (300 x 300 DPI)

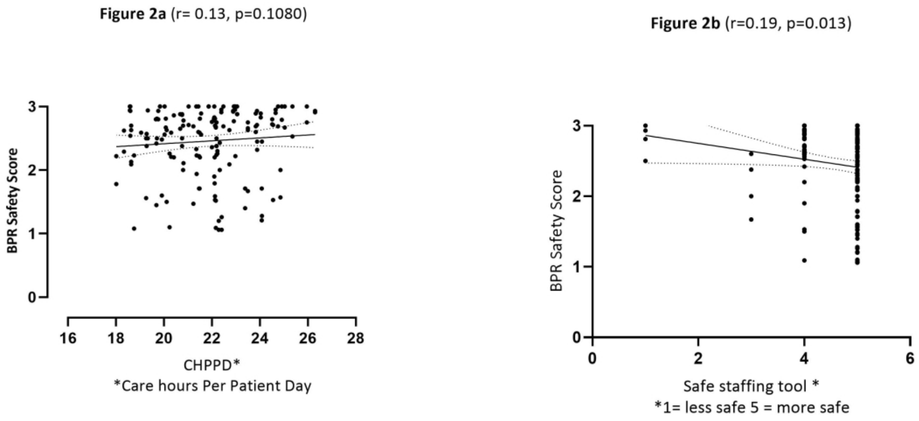


Figure 2a Relationship between bedside professional reported (BPR) shift safety and care hours per patient day (CHPPD) (n=170/ 174 shifts)
Figure 2b Relationship between bedside professional reported (BPR) shift safety (BPR) and safe staffing "score" 1=staff overutilization red, 5 = green staff underutilization (n=170/ 174 shifts)

252x119mm (300 x 300 DPI)

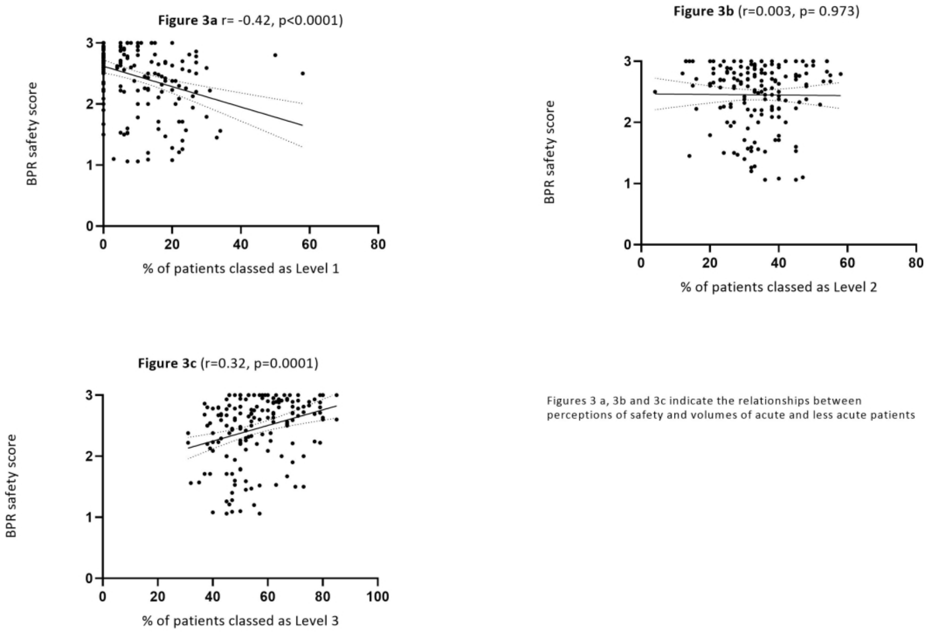


Figure 3 a, b & c Relationship between bedside professional reported (BPR) shift safety and volumes of acute and less acute patients Level 1, Level 2, Level 3 (n=170/ 174 shifts)

251x171mm (300 x 300 DPI)

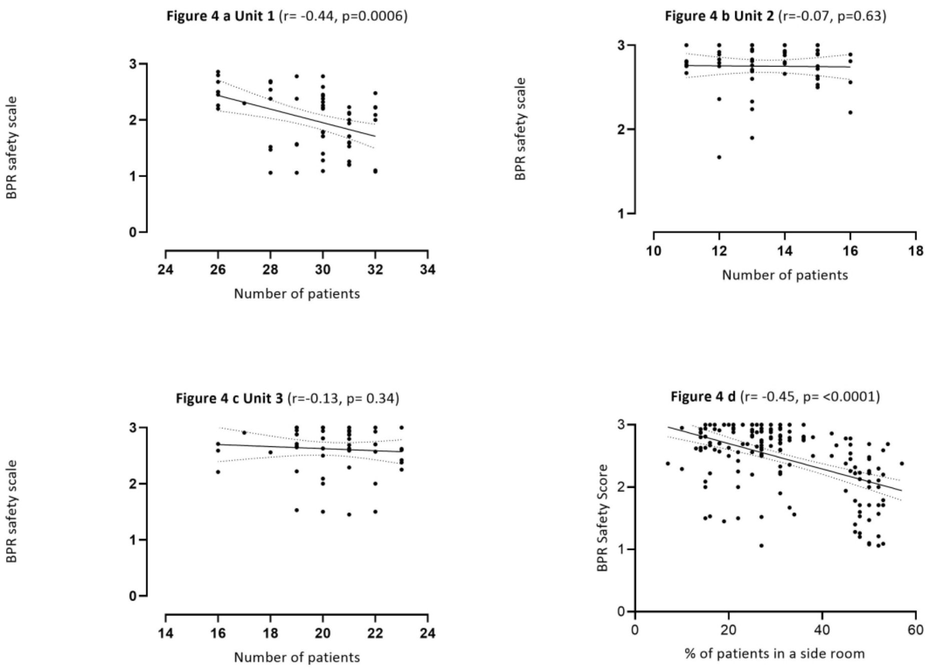


Figure 4 a, b & c Relationships between bedside professional reported (BPR) shift safety and number of patients in unit 1 (n=58), 2(n=56) and 3(n=56) Figure 4d Relationship between bedside professional reported perceptions of shift safety (BPR) and the percentage of patients in a side room (n=170/ 174 shifts).

252x180mm (300 x 300 DPI)

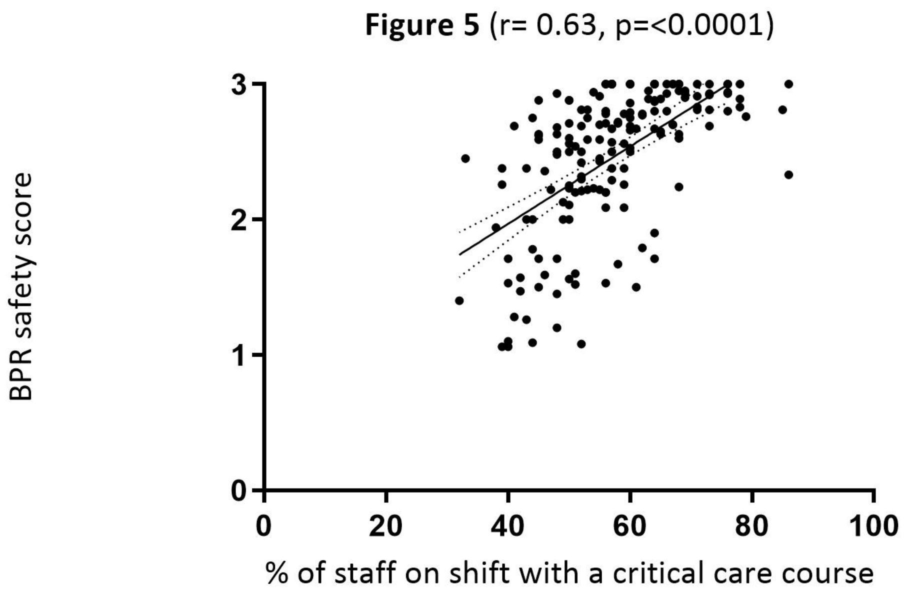


Figure 5 Relationship between bedside professional reported (BPR) shift safety and the percentage of staff on a shift with a critical care course (n=170/ 174 shifts).

124x80mm (300 x 300 DPI)

**SUPPLEMENTARY MATERIALS**

**Supplementary File**

**Supplementary Table 1 Department of Health 2009 Levels of Care classification**

| | |
|---|---|
| Level 0 | Patients whose needs can be met through normal ward care in an acute hospital |
| Level 1 | Patients at risk of their condition deteriorating, or those recently relocated from higher levels of care, whose needs can be met on an acute ward with additional advice and support from the Critical Care team |
| Level 2 | Patients requiring more detailed observation or intervention including support for a single failing organ system or post-operative care and those 'stepping down' from higher levels of care |
| Level 3 | Patients requiring advanced respiratory support alone, or basic respiratory support together with support of at least two organ systems. This level includes all complex patients requiring support for multi-organ failure. |

**Supplementary Table 2 Characteristics of ICU sites during data collection**

| Site | Number of available beds | Number of side rooms | Core Specialities |
|---|---|---|---|
| Site 1 | 32 | 16 | Major Trauma Centre, Vascular, General |
| Site 2 | 16 | 4 | Heart Attack Centre, Hepatobiliary, Renal and transplant, Haematology/Oncology |
| Site 3 | 24 | 5 | Stroke centre, Neurological, Neurosurgical, Head and Neck surgery, General. |

For peer review only

**Supplementary Table 3a Safer Nursing Care Tool**

| Level of Care | Descriptor |
|---|---|
| Level 0<br>Patient requires hospitalisation Needs met by provision of normal ward carers. | **Care requirements may include the following**<br>• **Elective medical or surgical admission**<br>• **May have underlying medical condition requiring on-going treatment**<br>• **Patients awaiting discharge**<br>• **Post-operative/post-procedure care - observations recorded half hourly initially then 4-hourly**<br>• **Regular observations 2 - 4 hourly**<br>• **Early Warning Score is within normal threshold.**<br>• **ECG monitoring**<br>• **Fluid management**<br>• **Oxygen therapy less than 35%**<br>• **Patient controlled analgesia**<br>• **Nerve block**<br>• **Single chest drain**<br>• **Confused patients not at risk**<br>• **Patient requires assistance with some activities of daily living, requires the assistance of one person to mobilise, or experiences occasional incontinence** |
| Level 1a<br>Acutely ill patients requiring intervention or those who are UNSTABLE with a GREATER POTENTIAL to deteriorate | **Care requirements may include the following**<br>• **Increased level of observations and therapeutic interventions**<br>• **Early Warning Score - trigger point reached and requiring escalation.**<br>• **Post-operative care following complex surgery**<br>• **Emergency admissions requiring immediate therapeutic intervention.**<br>• **Instability requiring continual observation/invasive monitoring**<br>• **Oxygen therapy greater than 35% +/- chest physiotherapy 2-6 hourly**<br>• **Arterial blood gas analysis - intermittent**<br>• **Post 24 hours following insertion of tracheostomy, central lines,epidural or multiple chest or extra ventricular drains**<br>• **Severe infection or sepsis** |
| Level 1b<br>Patients who are in a STABLE condition but are dependant on nursing care to meet most or all of the activities of daily living | **Care requirements may include the following**<br>• **Complex wound management requiring more than one nurseor takes more than one hour to complete.**<br>• **VAC therapy where ward-based nurses undertake the treatment**<br>• **Patients with Spinal Instability /Spinal Cord Injury**<br>• **Mobility or repositioning difficulties requiring the assistance of two people** |

For peer review only

| | |
|---|---|
| | • **Complex Intravenous Drug Regimes - (including those requiring prolonged preparatory /administration/post-administration care)**<br>• **Patient and/or carers requiring enhanced psychological support owing to poor disease prognosis or clinical outcome**<br>• **Patients on End of Life Care Pathway**<br>• **Confused patients who are at risk or requiring constant supervision**<br>• **Requires assistance with most or all activities of daily living**<br>• **Potential for self-harm and requires constant observation**<br>• **Facilitating a complex discharge where this is the responsibility of the ward-based nurse** |
| Level 2<br>May be managed within clearly identified, designated beds, resources with the required expertise and staffing level OR may require transfer to a dedicated Level 2 facility /unit | **Deteriorating/ compromised single organ system**<br>• **Post operative optimisation (pre-op invasive monitoring)/extended post-op care.**<br>• **Patients requiring non-invasive ventilation/respiratory support;**<br>**CPAP/BiPAP in acute respiratory failure**<br>• **First 24 hours following tracheostomy insertion**<br>• **Requires a range of therapeutic interventions including:**<br>• **Greater than 50% oxygen continuously**<br>• **Continuous cardiac monitoring and invasive pressure monitoring**<br>• **Drug Infusions requiring more intensive monitoring e.g. vasoactive drugs (amiodarone, inotropes, gtn) or potassium, magnesium**<br>• **Pain management - intrathecal analgesia**<br>• **CNS depression of airway and protective reflexes**<br>• **Invasive neurological monitoring** |
| Level 3<br>Patients needing advanced respiratory support and/or therapeutic support of multiple organs. | **Monitoring and supportive therapy for compromised/ collapse of two or more organ/ systems**<br>• **Respiratory or CNS depression/ compromise requires mechanical/invasive ventilation**<br>• **Invasive monitoring, vasoactive drugs, treatment of hypovolaemia/haemorrhage/ sepsis or neuro protection** |
| | |

For peer review only

**Supplementary Table 3b**

| **SafeCare Allocate Colour** | **SafeCare Allocate Calculation** | **Category for analysis** |
|---|---|---|
| Green | 90% (underutilisation ) | 5 |
| None given | 90.1%-104.9% | 4 |

| | | |
|---|---|---|
| Amber | 105 % (overutilisation) | 3 |
| None Given | 105.1%-109.9% | 2 |
| Red | 110% (overutilisation) | 1 |

**Supplementary Table 4 Summary of Responses**

| | Total | | Site 1 | | Site 2 | | Site 3 | |
|---|---|---|---|---|---|---|---|---|
| | Total | % | Total | % | Total | % | Total | % |
| Green | 1731 | 61% | 495 | 39% | 685 | 83% | 551 | 74% |
| Amber | 523 | 18% | 304 | 24% | 101 | 12% | 118 | 16% |
| Red | 582 | 21% | 461 | 37% | 44 | 5% | 77 | 10% |
| Total responses | 2836 | | 1260 | | 830 | | 746 | |
| Response rate | 57.7% | | 56.4% | | | 70.8% | | 46.0% |

For peer review only

**Supplementary Table 5** Analysis of relationships between BPR and staffing, patient and work environment characteristics using mean, median and weighted mean BPR score.

| Variable tested | BPR Score Mean* | | BPR Score Median** | | BPR Score Mean weighted *** | |
|---|---|---|---|---|---|---|
| | r value | p value | r value | p value | r value | p value |
| **CHPPD** | 0.13 | 0.108 | 0.01 | 0.885 | -0.11 | 0.151 |
| **Safe care Allocate Score** | -0.19 | 0.013 | -0.17 | 0.032 | 0.17 | 0.032 |
| **% of level 1 patients** | -0.42 | <0.0001 | -0.34 | <0.0001 | 0.41 | <0.0001 |
| **% of level 2 patients** | 0.003 | 0.973 | 0.02 | 0.78 | -0.0004 | 0.996 |
| **% of level 3 patients** | 0.32 | <0.0001 | 0.22 | 0.004 | -0.32 | <0.0001 |
| **Number of patients** | -0.6 | <0.0001 | -0.54 | <0.0001 | 0.59 | <0.0001 |
| **% of patients in side rooms** | -0.45 | <0.0001 | -0.39 | <0.0001 | 0.44 | <0.0001 |
| **% of staff with a CCC** | 0.63 | <0.0001 | 0.55 | <0.0001 | 0.61 | <0.0001 |

***each green response scores 3, each amber response scores 2, each red response scores 1, total score divided by the number of responses= BPR shift score. (3=safe 1 = very unsafe)**
****each green scores 3, each amber scores 2, each red scores 1, median score used= BPR shift score (3 =safe 1 =very unsafe)**
***** each green response scores 1, each amber scores 3, each green response scores 5, total score divided by the number of responses= BPR shift score (1= safe 5 = very unsafe)**

For peer review only

**Supplementary Figure 1**

Image 1

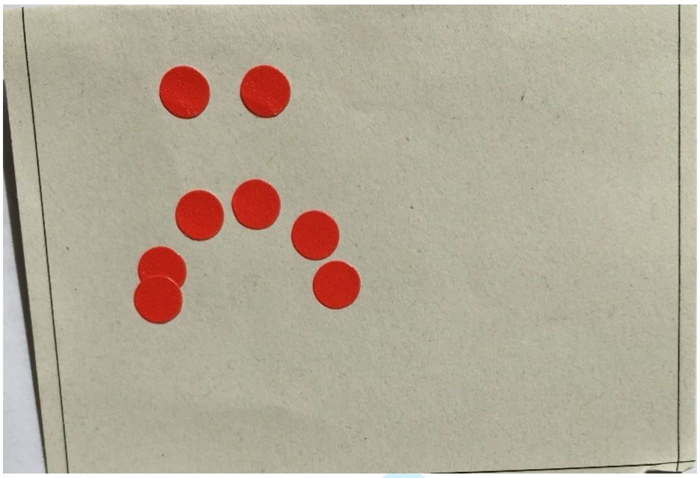

For peer review only

For peer review only

BMJ Open

# An exploratory study of staff perceptions of shift safety in the critical care unit and routinely available data on workforce, patient and organisational factors.


| | |
|---|---|
| Journal: | *BMJ Open* |
| Manuscript ID | bmjopen-2019-034101.R1 |
| Article Type: | Original research |
| Date Submitted by the Author: | 19-Mar-2020 |
| Complete List of Authors: | Leon-Villapalos, Clare; Imperial College Healthcare NHS Trust, Critical Care<br>Wells, Mary; Imperial College London; Imperial College Healthcare NHS Trust, Directorate of Nursing<br>Brett, Stephen; Imperial College London, Surgery and Cancer; Imperial College Healthcare NHS Trust, Critical Care |
| <b>Primary Subject Heading</b>: | Intensive care |
| Secondary Subject Heading: | Health services research |
| Keywords: | Adult intensive & critical care < ANAESTHETICS, Safety, Staff deployment |
| | |


SCHOLARONE™
Manuscripts

*I, the Submitting Author has the right to grant and does grant on behalf of all authors of the Work (as defined in the below author licence), an exclusive licence and/or a non-exclusive licence for contributions from authors who are: i) UK Crown employees; ii) where BMJ has agreed a CC-BY licence shall apply, and/or iii) in accordance with the terms applicable for US Federal Government officers or employees acting as part of their official duties; on a worldwide, perpetual, irrevocable, royalty-free basis to BMJ Publishing Group Ltd ("BMJ") its licensees and where the relevant Journal is co-owned by BMJ to the co-owners of the Journal, to publish the Work in this journal and any other BMJ products and to exploit all rights, as set out in our licence.*

*The Submitting Author accepts and understands that any supply made under these terms is made by BMJ to the Submitting Author unless you are acting as an employee on behalf of your employer or a postgraduate student of an affiliated institution which is paying any applicable article publishing charge ("APC") for Open Access articles. Where the Submitting Author wishes to make the Work available on an Open Access basis (and intends to pay the relevant APC), the terms of reuse of such Open Access shall be governed by a Creative Commons licence – details of these licences and which Creative Commons licence will apply to this Work are set out in our licence referred to above.*

*Other than as permitted in any relevant BMJ Author's Self Archiving Policies, I confirm this Work has not been accepted for publication elsewhere, is not being considered for publication elsewhere and does not duplicate material already published. I confirm all authors consent to publication of this Work and authorise the granting of this licence.*

For peer review only

**TITLE PAGE**

**Title of the article: An exploratory study of staff perceptions of shift safety in the critical care unit and routinely available data on workforce, patient and organisational factors.**

Clare Leon-Villapalos [(1)], Mary Wells [(2)], Stephen J Brett [(3)]

[(1)] Corresponding author Clare Leon-Villapalos, Intensive Care Unit, Imperial College Healthcare NHS Trust. 9th Floor QEQM St. Mary's Hospital, Praed Street London W21NY clare.leon-villapalos@nhs.net Telephone 02033126666.

[(2)] Professor Mary Wells, Professor of Practice (Cancer Nursing), Imperial College London and Lead Nurse for Research, Directorate of Nursing, Imperial College Healthcare NHS Trust. London. UK.

[(3)] Professor Stephen J Brett. Professor of Critical Care, Dept. of Surgery and Cancer, Imperial College London, Consultant in Intensive Care Medicine, Imperial College Healthcare NHS Trust. London. UK.

Word count: 3956

Author ORCID ID

CLV 0000-0002-1610-0319

MW 0000-0001-5789-2773

SJB 0000-0003-4545-8413

**Keywords**

- Safety
- Critical care
- Staff deployment

For peer review only

## ABSTRACT

**Objectives**

To explore: bedside professional reported (BPR) perceptions of safety in intensive care staff; and the relationships between BPR safety, staffing, patient and work environment characteristics.

**Design** An exploratory study of self-recorded staff perceptions of shift safety and routinely collected data.

**Setting** A large teaching hospital comprising 70 critical care beds.

**Participants** All clinical staff working in adult critical care.

**Interventions** Staff recorded whether their shift felt "safe, unsafe or very unsafe" for 29 consecutive days. We explored these perceptions and relationships between these and routine data on staffing, patient and environmental characteristics.

**Outcome measures** Relationships between BPR safety and staffing, patient and work environment characteristics.

**Results** 2836 BPR scores were recorded over 29 consecutive days (response rate 57.7%). Perceptions of safety varied between staff, including within the same shift. There was no correlation between perceptions of safety and two measures of staffing; care hours per patient day (r= 0.13 p=0.108) and Safecare Allocate (r= -0.19 p=0.013). We found a significant, positive relationship between perceptions of safety and the percentage of Level 3 (most severely ill) patients (r=0.32, p=0.0001). There was a significant inverse relationship between perceptions of safety and the percentage of Level 1 patients on a shift (r= -0.42, p=<0.0001). Perceptions of safety correlated negatively with increased numbers of patients (r= -0.44, p=0.0006) and higher percentage of patients located side rooms (r=0.63, p<0.0001). We found a significant relationship between perceptions of safety and the percentage of staff with a specialist critical care course (r=0.42. p=0.0001).

**Conclusion** Existing staffing models, which are primarily influenced by staff:patient ratios may not be sensitive to patient need. Other factors may be important drivers of staff perceptions of safety and should be explored further.


**Trial registration**- UK Health Research Authority approval was obtained (ID249248).

## STRENGTHS AND LIMITATIONS OF THE STUDY

- Our study included staff from all professions working in critical care.
- The study achieved a fair response rate from staff with complete information on patient status and organisational metrics.
- This exploratory study was from different intensive care units, but from the same overarching institution- thus we cannot be certain how far our observations can be generalised.
- In this study we were unable to explore the meaning behind why staff felt a particular shift was "safe" or not.

**FUNDING STATEMENT**

This work was supported by Imperial Health Charity, National Institute of Health Research (NIHR) Imperial Biomedical Research Centre funded predoctoral research grant (grant number RF18\100007).

**COMPETING INTERESTS**

The authors have no associations with commercial entities that provided support for the submitted manuscript or any financial or non-financial associations relevant to the submitted manuscript.

**INTRODUCTION**

Intensive care units (ICUs) are complex and expensive services where staffing is the greatest cost. Inadequate staffing can have serious consequences, including failure to provide fundamental care and patient harm. (1, 2) Research suggests there is an interdependent relationship between the nature and composition of the workforce, work environment and patient outcomes including mortality, in both acute, (3-9) and ICU (10-12) settings. The mechanisms that mediate these relationships are incompletely understood. (13) High quality, patient-centred care requires appropriately skilled and available personnel operating within staffing models that optimise their performance, but this may be difficult to achieve in the context of fiscal and labour market challenges. (13,14)

ICU specific staffing guidance, such as the United Kingdom Guidelines for the Provision of Intensive Care Services (15), is primarily based on staff:patient ratios, and the 'severity' of illness, as defined by the number of organs in failure, (16) (Supplementary table 1). The nature and intensity of critical care is, however, changing in response to multiple drivers including; technological advances, more numerous and more complex patients, an increased focus on survivorship and rehabilitation (17) and changing expectations of patients and families, therefore more complex models for staff deployment may be required.

Current approaches to safe staffing may not be sensitive to actual patient need or to rapid or unpredictable fluctuations in workload. Furthermore, it is unclear how tools to guide staffing establishments translate into staff deployment and staff *perceptions* of care. ICU workforce guidance (15) also tends towards a uni-professional "siloed" approach that does not reflect the current multi-professional nature of intensive care (18) or the significance of inter-professional dynamics for productivity. (19,20)

The degree to which existing models of staffing in ICU reflect issues of relevance to staff is also unclear, specifically, staff *perceptions* of "safety"- a term used colloquially to reflect an environment, human and physical, which minimises the risk of avoidable adverse events and deficits in care. Recent studies have indicated that perceptions of safety vary within institutions, (21) between individuals (22) and between staff and directors of institutions. (21) Perceived safety may also contribute to staff stress- particularly when there is a discrepancy between expectations of resource and actual resources, including staffing. (23, 24) Staff stress is recognised as prevalent and concerning (25) in the ICU workforce and may contribute to reduced quality of care, burnout and poor staff retention. (26)

Relationships between staff *perceptions* of "safety" and shift by shift variations in staffing metrics, staff deployment, patient and unit factors have not been explored in ICU. There have been few

For peer review only

studies which examine the contribution and perspectives of all healthcare professionals within ICU, although this has been identified as a priority. (11,27).

This study therefore sought to contribute to our understanding of the complexity of staffing in ICU by:

Recording staff grading of perceived safety, on a shift by shift basis, using a bedside professional reported (BPR) safety estimate (for all professions).

Exploring the relationships between BPR safety and routinely available data on; staffing, patient and work environment factors that influence deployment.

## METHODS.

This study was performed at Imperial College Healthcare NHS Trust, across 70 general critical care beds. The three units vary in speciality, size and number of side rooms (Supplementary Table 2) but are part of one institution with shared processes, staffing and staff deployment models . UK Health Research Authority approval was obtained (ID249248). No patient or staff identifiable data were used, and explicit consent was not required. Data were collected over 29 consecutive days during October and November 2018. During the study period each site was visited or contacted daily by one of the investigators (CLV), to collect and verify data.

### Patient and Public Involvement

There were no funds or time allocated for patient or public involvement.

The target population, staff from all professional groups, were involved in informal discussion of the research question, directly participating in the study and in ‘sense checking’ the methods of analysis. Results of the study were disseminated to several staff forums, including those for people in leadership positions who direct staffing deployment.

### Data collection and measures

#### Bedside Professional Rating of Safety (BPR).

We asked all ICU staff to rate each shift as “safe, unsafe, or very unsafe” using a coloured sticker (green, amber or red). This question was generated from previous work into staff experiences of safety. (22) During staff briefings and daily visits to sites, participants were encouraged to interpret the term “safe” as their own perception of safe in its normal conversational sense in ICU.

Staff were asked to choose one sticker to rate each shift and put this onto a card labelled with the relevant date and shift (e.g. October 4th Night shift). Participants posted the cards in data collection boxes, allowing them to complete the process anonymously, thus we were not able to identify participants by profession.

To allow us to look for relationships between perceptions of safety and other variables, including staffing, patient and work environment factors, a BPR shift score was created for each shift using the following method; we counted the responses and allocated a score of 1 for each red response, 2 for each amber response and 3 for each green response. We summed the total score and divided this by the number of responses for that shift. This

For peer review only

created a mean shift safety score *between* 1 (very unsafe) and 3 (safe), which reflected the diversity and weighting of responses and is simple to reproduce. This method was discussed with senior nursing staff to confirm that they felt it reasonably summarized perceptions of safety on a given shift.

**Routinely available data on staffing, patient and work environment**

**Staffing and workload data.**
We recorded the number of staff (including nurses, doctors and allied health professionals) working clinically in the ICU during the study period. Staffing data were extracted from an electronic rostering system and verified with the nurse in charge. Where professional groups did not use electronic rostering e.g. physiotherapists, the data were collected daily and confirmed weekly with the relevant manager. Staff who worked clinically for part of a 12-hour shift were represented as a proportion of a whole-time equivalent (e.g. a physiotherapist working for 6 hours on ICU was counted as 0.5).

We calculated workload intensity scores by summing the total number of organs in failure for each day and dividing this by the number of staff working that day. We looked at this by profession and by all professions combined. We used data from the critical care minimum data set (CCMDS) (28) to calculate the number of organs each patient had in failure, this was only available by 24-hour period, and therefore, data were collapsed into a 24-hour period.

We recorded the care hours per patient day (CHPPD) from our electronic roster. CHPPD is a measure that can be used to assess productivity and facilitates benchmarking. (29) We recorded staffing utilization data from SafeCare in the commercial rostering system provided by Allocate (AllocateSoftware Richmond 2017). This uses the composite acuity/dependency scoring system from the Safer Nursing Care Tool (30) (Supplementary Table 3a) to provide nursing utilisation reports to support real-time deployment decisions. This is done by calculating required nursing hours (based on acuity and dependency data entered by senior nursing staff) and dividing this figure by the number of rostered nursing hours to produce a percentage of nursing hours utilization (e.g. required hours 126.4, rostered hours 115.00 = 109.9 % utilisation).
This figure is reported in the following (partially) colour coded categories: : <90% Green, 90.1% -105.1% %, 105% Amber, 105.1%- 110%, 110.1% Red. There is meaning attached to these five categories, so that green is reported as underutilisation whilst all figures falling into amber and above are reported as overutilisation. In our analysis we reflected these categories by allocating a number to each so that green = 5 through to red =1. This is illustrated in detail in (Supplementary Table 3b).

During the study if we found within-shift changes in staff numbers, patient numbers or patient acuity we recorded the status at approximately 5:00 and 17:00 (day shifts). This was a pragmatic decision that reflected the timings of local processes to manage staffing.

**Patient characteristics data.**

Data regarding the number of Level 1,2 and 3 patients (15) were recorded by visiting or contacting each site daily. In ICU, patients are broadly categorised by the number of organs in failure, with Level 3 patients being the most severely ill (Supplementary table 1).

**Work environment data.**
We recorded the total number of patients and the number of patients in side rooms per shift and confirmed this daily with a senior nurse.

For peer review only

**Data regarding nurse training.**
We recorded the number of nursing staff on each shift who had completed a post registration critical care course (CCC), the accuracy of the data were confirmed with local clinical nurse educators. We did not collect staff characteristic data for other professional groups as this is not routinely available, and the smaller numbers of the group sizes would impact on our ability to perform statistical analysis

## Analysis

Data were collated into an Excel spreadsheet (Microsoft, Redmond, Washington State, USA). We used descriptive statistics to analyse frequency of responses. Data were tested for normality using the D'Agostino-Pearson Omnibus test and non-parametric tests were used where appropriate. We used Spearman's rank correlation to evaluate strength and direction of relationships; regression lines and 95% confidence intervals were plotted for illustrative purposes. Prism (v8.01 for Windows, GraphPad Software, La Jolla California USA) was used throughout.
Although the shift BPR was primarily analysed as a mean for simplicity – we further explored this using both median and a weighted approach (whereby a weighting of 5 was given to very unsafe, 3 to unsafe and 1 to safe). The purpose of this was to allow us to explore the sensitivity of the BPR tool.

## RESULTS

### Exploring BPR perceptions of safety

A total of 2836 BPR responses were received during 29 days of data collection at 3 sites, we received responses from 170 out of a total of 174 twelve-hour shifts (98% of shifts). The overall response rate, using a denominator of all ICU staff (that is all staff working on ICU, including those who were transient or only partially based on ICU) was 57.7 %. The combined responses are illustrated in Figure 1a. Most responses were green "safe" (61%), 18 % of responses were amber, "unsafe" and 21% of responses were red, "very unsafe". Total responses and responses by site are illustrated in Supplementary Table 4.
We noted that staff perceptions frequently varied within the same shift; for only 13% of shifts did all staff who responded report the same perception of safety for their site.
We calculated a BPR shift safety rating, using the methods described previously, which summarised general shift responses and allowed us to look for relationships between reported shift safety and staffing, patient and work environment characteristics. The range of mean shift BPR scores was 1.00 - 3.00, median 2.63. The distribution of the BPR shift scores for 170 shifts is illustrated in Figure 1b.

The BPR tool summarizes the overall feeling of a shift by incorporating all responses but does not differentiate between shifts with low and high response rates. To address this we looked to see if there was a relationship between response rates and perceptions of safety which might reflect a systematic bias, we found a weak relationship between staff perceiving a shift to be safe and a higher response rate ($r = 0.156$, $p = 0.0426$ Figure 1 c).

For peer review only

**Perceptions of safety and staffing data.**

We did not find a significant relationship between perceptions of safety and CHPPD (r= 0.13 p=0.1080, Figure 2a). We found a weak inverse relationship between perceptions of safety and staffing utilization (SafeCare Allocate) (r = -0.19, p = 0.013 Figure 2b).
We found a very weak relationship between perceptions of safety and workload "intensity" (defined as number of organs in failure/total number of nurses on shift). Looking only at nursing hours, staff reported feeling safer at higher rather than lower intensity workloads r= 0.15 p=0.047). We did not find a significant relationship between these variables when we included doctors' hours (r =0.14 p=0.73) and allied health professionals (AHP) hours (r = 0.13, p=0.09).

**Perceptions of safety and patient characteristics**

We found a significant, inverse relationship between perceptions of safety and the percentage of Level 1 patients on a shift (p=<0.0001, r= -0.42 Figure 3a). There was no significant relationship between perceptions of safety and the percentage of Level 2 patients (p=0.9729, r= 0.003 Figure 3b). We found a significant, positive, relationship between perceptions of safety and the percentage of Level 3 patients (p=0.0001, r= 0.32 Figure 3c).

**Perceptions of safety and the work environment**

We found a significant inverse relationship between perceptions of safety and numbers of patients in the largest unit (Unit 1) where number of patients ranged between 27 and 32 (figure 4a r=-0.44, p=0.0006). We did not find a significant relationship in the smaller units (where numbers of patients were between 11-16 and 17-23 respectively figures 4b & c). We also found a significant inverse relationship between perceptions of shift safety and the percentage of patients who were occupying side rooms in a given shift (r= -0.45 P<0.0001 Figure 4d).
We found a significant, positive relationship between the percentage of nursing staff who had a critical care course on each shift and perceptions of safety (r = 0.63, p= <0.0001 Figure 5). We also looked at day versus night scores and there was robustly no difference between perceptions of safety .

These analyses were repeated using both median scores and a score that gave additional weighting to "unsafe" responses, we found only minor differences in the relationships using these methodologies. These are summarised in Supplementary table 5.

**DISCUSSION**

Our study sought to explore an estimate of staff *perceptions* of shift safety for all professions in ICU on a shift by shift basis; we termed this bedside professional reported (BPR) safety. We also explored the relationships between this estimate and other factors, including routinely available data on staffing, patient and work environment factors that influence deployment. Staff perceptions of safety are important as they may reflect safety culture (31) and be associated with the risk of adverse events (32) including risks not identified by organisational processes. (1) We observed that staff found the BPR tool easy to implement and seemed acceptable to use (although no formal assessment of acceptability was made); previous pilots using more ambitious methods had proved unsuccessful due to poor engagement. The BPR's simplicity and fair completion rates are strengths and are important features of safety tools (31); it also yields unit level data, which could be used to inform local discussion and action. We are not aware of any studies of similar tools in ICU, and although further validation of the BPR tool is needed, the relationships we found were plausible. The very weak relationship we found between higher response rates and perceived safety indicates

that we cannot rule out a systematic bias, with higher response rates on “safer” shifts. However, neither the strength or direction of the relationship is pronounced. Exploring the data using a median -based or weighted approach to BPR did not alter the observed relationships (Supplementary Table 5). Organisations wishing to create a more safety sensitive system might consider further adjustments to weighting.

For most shifts during the study period we found heterogeneity in reported perceptions of safety. Similarly, different “risk horizons” between and within staff groups on ICU, depending on team and environmental factors, have been previously described. (22). This may support an argument that training for those with leadership roles in ICU should explicitly address heterogeneity of experience, perceptions of safety and anxiety amongst ICU staff.

Staff recorded their *perceptions* of shift safety; being involved in an enterprise which is not felt by the individual to be “safe” may be a source of stress. This was reflected in the way some staff responses seemed to be framed (which included annotations and pictures, Supplementary Figure 1). We found during data collection, that staff described the stickers as “cathartic” suggesting that the BPR facilitated the articulation of feelings that were not captured elsewhere. It is reasonable to suggest that being able to communicate concerns about safety to someone who will listen may be important for staff wellbeing and trust in leadership. This may be of particular importance in ICU, an environment where staff stress and burnout are areas of concern. (25,33)

Perceptions of shift safety were not well correlated with some nursing staffing metrics (NB deployment metrics for AHPs and medical staff are not currently in use at our organisation). The lack of relationship between these organisational measures and staff perceptions reinforces the importance of triangulating staff deployment decisions with professional judgment and suggests organisations should be cautious in relying solely on mathematically derived models. This may be one of the reasons that CHPPD is not consistently accepted by Nurse Directors (34) and it is already noted that SafeCareAllocate has not been validated for use in deployment. (35) Although the use of staffing benchmarks to prevent the erosion of staffing numbers is likely to remain important, the findings of this study suggest that more sophisticated thinking is needed to inform staffing models.

Our study found a significant relationship between patient acuity and perceptions of safety. Perhaps counterintuitively, more staff reported feeling safe when there were larger numbers of severely unwell (Level 3) patients, and reported feeling shifts to be “unsafe” when there were larger numbers of low acuity patients (Level 1). We did not specifically explore what mediated this relationship, but possible explanations include: that existing ICU staff deployment models are more sensitive to need for high acuity than for lower acuity patients; that staff are less confident in caring for this patient group; or that they are a marker for other workflow stressors, such as throughput or a very dynamic workload. This finding may indicate that uni-professional approaches that allocate fewer nursing hours to less severely unwell patients (where severity of illness based on categories of organ failure) may not match staff perceptions of patient need.

We found that size of unit and an increased proportion of patients located in side rooms were significantly related to staff reporting the shift as feeling less safe. We did not explore what mediated these relationships; possible explanations might include the impact of larger volumes of patients on individuals’ capacity so that there may be a degree of activity which is beyond most staff’s comfort zone. In the largest unit, processes were starting to be put in place to organise this as two smaller units but these were not fully embedded during the study period. Side rooms are increasingly common in ICU due to the benefits of improved patient privacy, confidentiality and

For peer review only

infection control. However, there is little research into the impact on staff. Recent studies conducted in acute wards suggest that trade-offs for nursing staff include increased staff anxiety due to decreased surveillance and fewer opportunities for informal education, role modelling and interaction. (36,37) Our findings suggest that there may be value in exploring the relationships between staff perceptions of safety and unit design to identify modifiable factors, as larger units and side rooms become increasingly common features of ICU's.

The strong positive relationship between perceptions of safety and the percentage of staff on each shift with a CCC supports guidance that this should be a benchmarked target (15) and suggests that there may be value in training local establishments to a level which enables this to be achieved in daily staffing rotas. Our finding is consistent with the evidence that increased nursing education (to Bachelor level) is associated with a reduced likelihood of patients dying (4). It is also supported by recent findings (38), which indicated that strain and burnout in ICU nurses is not inevitable and could be modified by an intensive education program which included situational role play, simulation and debriefing.

**LIMITATIONS**

Our study had several limitations:

Whilst the study included three separate units, it was conducted at a single NHS Trust. Findings therefore may reflect factors unique to our culture and organisation, however the volume of observations and range of specialities suggest that our data may have broader applicability. We did not perform subgroup analysis which may have yielded differences between units as the smaller groups would have been insufficient for statistical analysis.

This was an exploratory study and the first use of the BPR scale, which although not formally "validated" seemed to have face validity with participants and senior staff. The intensity-derived data might not be sensitive to variations within a given shift or between sites. Furthermore, although it seems intuitively obvious, we cannot know for certain that working on a shift experienced as "unsafe" causes anxiety. We did not attempt to capture anxiety because in a preliminary unpublished study we found that attempting to acquire too much data at the end of a busy shift produced poor survey return and we were keen to avoid undermining the primary objective.

The question "How safe was your shift" was taken from associated work undertaken previously on our unit. The initial unpublished pilot was perceived as too complex and did not gain traction; we therefore reduced the original 5-point response options to "safe, unsafe and very unsafe". During the data collection we noted that staff often annotated their responses with extra stickers, drawings or comments, indicating that three responses did not always allow them to express fully the depth or range of feelings- which seemed more important at the "unsafe" end. Equally it could be argued that there is no meaningful difference between unsafe and very unsafe and that these categories could be collapsed, we therefore felt the option we selected was reasonable.

Early conversations with staff indicated that many staff did not want to indicate their professional group when reporting their perception of shift safety, as they felt this might compromise anonymity, we therefore did not ask for this information. This meant we were not able to analyse responses by professional group. Whilst this information would have brought valuable insights, it may have

For peer review only

impacted on the response rates. Subsequent informal discussions have suggested that, having gained confidence in the tool and motivation behind the research project, staff would be willing to indicate their profession in future studies.

Despite a satisfactory response rate, we did not receive responses from all staff and therefore do not know whether the perceptions of staff who did not engage are different to those who did.

Most significantly, we do not know what lay behind different individual feelings of safety- or what might mediate the relationships we identified between perceptions of safety and the characteristics discussed. Further qualitative work is currently being undertaken to address this.

**IMPLICATIONS**

Understanding how to deploy the multi-professional team in a way that sensitively matches patient need in ICU is challenging and there is limited evidence available to inform guidance. Incorporating staff perceptions may be useful organisationally and may help identify modifiable factors that impact on staff stress and burnout.

The findings of our exploratory study suggest that

- A BPR tool is simple to use and seems acceptable to staff; it may capture near real-time staff perceptions of safety.
- Perceptions of safety vary between individuals on the same shift and this should be factored into the way teams are trained and led.
- Perceptions of safety may not align to some organisational staffing metrics, reinforcing the need to augment these with professional judgement and other measures.
- The composition of the workforce is important to staff perceptions of safety and our findings support the provision of specialist post-registration critical care course training for nursing staff.
- Further research could usefully focus on staff deployment for patients classified as less severely unwell and the impact of the size and layout of ICUs.

To our knowledge this is the first study to include allied health professionals in addition to nurses and doctors to explore perceptions of safety and staff deployment in ICU. This approach may yield results that better reflect the reality of how care is delivered in ICU and can also accommodate emerging roles such as nursing associates and advanced critical care practitioners. This study has relevance nationally; the factors explored reflect, or are similar to, guidance followed by most UK ICUs. The findings may be of interest outside of the UK since, whilst processes vary, there are shared concerns regarding workforce deployment, supply, costs and burnout.

**Legends for figures**

**Figure 1a** Bedside professional reported (BPR) safety October 4$^{th}$-November 2$^{nd}$ (n=2836).

For peer review only - http://bmjopen.bmj.com/site/about/guidelines.xhtml

**Figure 1b** Distribution of bedside professional reported (BPR) safety - shift scores (n=170/ 174 shifts).

**Figure 1 c** Relationship between bedside professional reported (BPR) safety and response rates (n=170/ 174 shifts).

**Figure 2a** Relationship between bedside professional reported (BPR) shift safety and care hours per patient day (CHPPD) (n=170/ 174 shifts)

**Figure 2b** Relationship between bedside professional reported (BPR) shift safety (BPR) and safe staffing "score" 1=staff overutilization red, 5 = green staff underutilization (n=170/ 174 shifts)

**Figure 3 a, b & c** Relationship between bedside professional reported (BPR) shift safety and volumes of acute and less acute patients Level 1, Level 2, Level 3 (n=170/ 174 shifts)

**Figure 4 a, b & c** Relationships between bedside professional reported (BPR) shift safety and number of patients in unit 1 (n=58), 2(n=56) and 3(n=56), (n=58)

**Figure 4d** Relationship between bedside professional reported perceptions of shift safety (BPR) and the percentage of patients in a side room (n=170/ 174 shifts).

**Figure 5** Relationship between bedside professional reported (BPR) shift safety and the percentage of staff on a shift with a critical care course (n=170/ 174 shifts).

**Contributorship Statement**

We are fully satisfied that all authors fulfill the criteria of authorship and that no-one else who fulfills the criteria has been excluded as an author.

C L-V and SJB conceived the idea for the study.

All authors contributed to the funding application, design and execution of the study.

CL-V, SJB and MW co-designed and planned the methodological approach to data collection, use of routine data and statistical analysis.

C L-V collected the data and used the statistical package PRISM to analyse data, with close involvement from SJB and MW, who helped make decisions about the appropriate statistical tests and processes to use.

All authors (CLV ,SJB and MW) contributed to the review, analysis and interpretation of data.

All authors (CL-V, MW and SJB) discussed and decided upon the focus and content of the paper and the presentation of results.

C L-V wrote an initial draft of the paper which was then revised critically and re-drafted by all authors (CL-V MW and SJB).

CL-V, SJB and MW all made substantial contributions to the design, analysis, interpretation and writing up of this study, and have approved the final manuscript.

All authors (CL-V, SJB AND MW) agree to be accountable for all aspects of the of the work

For peer review only

**Acknowledgements**

**Funding Statement**

This work was supported by Imperial Health Charity, National Institute of Health Research (NIHR) Imperial Biomedical Research Centre funded predoctoral research grant (grant number RF18\100007).

**Competing Interests**

The authors have no associations with commercial entities that provided support for the submitted manuscript or any financial or non-financial associations relevant to the submitted manuscript.

**Data sharing statement**

Extra data can be accessed via the Dryad data repository at http://datadryad.org/ with the doi: 10.5061/dryad.v9s4mw6s7

**REFERENCES**

1 Francis R. The Mid Staffordshire NHS Foundation Trust Public Inquiry. Report of the Mid Staffordshire NHS Foundation Trust Public Inquiry: Executive Summary. London: Stationery Office, 2013.

2 Berwick D.A promise to learn- a commitment to act: improving the safety if patients in England. (2013) London: Department of Health.

3 Griffiths P, Maruotti A, Recio Saucedo A, *et al.* Nurse staffing, nursing assistants and hospital mortality: retrospective longitudinal cohort study. *BMJ Qual Saf*;**28:**609-617. doi:10.1136/bmjqs-2018-008043.

4 Aiken LH, Sloane DM, Bruyneel L *et al*. Nurse Staffing and education and hospital mortality in nine European countries: a retrospective observational study. *The Lancet* 2014;**383**:1824-30. doi:10.1016/S0140-673(13)62631-8.

5 Aiken LH, Sloane D, Griffiths P, *et al*. Nursing skill mix in European hospitals: cross-sectional study of the association with mortality, patient ratings, and quality of care. *BMJ Qual Safety*2016*;***26**: 559-568doi: 10.1136/bmjqs-2016-005567.

6. Griffiths P, Ball J, Murrells T, *et al*. Registered nurse, healthcare support worker, medical staffing levels and mortality in English hospital trusts: a cross-sectional study. *BMJ Open* 2016;**6**:e008751.doi10.1136/bmjopen-2015-008751.

7 Needleman J, Buerhaus P, Pankratz VS, *et al.* Nurse staffing and inpatient hospital mortality. *N Engl J Med*2011;**364:**1037-45. doi:10.1056/NEJMsa1001025.

8 Rafferty AM, Clarke S, Coles J *et al*. Outcomes of variation in hospital nurse staffing in English hospitals: Cross-sectional analysis of survey data and discharge records. *Int J Nurs Stud*2007;**44** (2):175-182 doi: 10.1016/j.ijnurstu.2006.08.003.

For peer review only

9 Aiken L, Sloane D, Clarke S *et al* Importance of work environments on hospital outcomes in nine countries *Int J for Quality in Healthcare* 2011; 23(4):357-364 doi.org/10.1093/intqhc/mzr022

10 Provonost PJ, Dang D, Dorman T *et al* Intensive care unit staffing and the risk for complications after abdominal aortic surgery. *Eff Clin Pract*2001;**4**:199-206.

11 West E, Baron DN, Harrison D *et al*. Nurse staffing, medical staffing and mortality in intensive care: an observational study. *Int J Nurs Stud*2014; **51:**781-94. doi:10.1016/j.ijnurstu.2014.02.00.

12 Hugonnet S, Uckay I & Pitter D. Staffing level: a determinant of late-onset ventilator-associated pneumonia. *Crit Care*2007;11 doi 10.1186/cc5974.

13 Leary A, Punshon G. Determining acute nurse staffing: a hermeneutic review of an evolving science. *BMJ Open*2019; **9**:e025654 doi 10.1136/bmjopen-2018-025654.

14 Borneo A. Staffing for Safe and Effective Care Nursing on the Brink. RCN Policy Report. 2018. London.

15 The Faculty of Intensive Care Medicine. Guidelines for the Provision of Intensive Care Services version 2. 2019 https://www.ficm.ac.uk/standards-research-revalidation/guidelines-provision-intensive-care-services-v2 accessed June 2019.

16 Department of Health. Comprehensive Critical Care: A Review of Adult Critical Care Services. 2000.

17 National Institute for Healthcare Excellence. Rehabilitation after critical illness in adults. Clinical guideline [CG83]. March 2009.

18 Donovan A Matthew Aldrich J Kendall Gross et al. Interprofessional care and teamwork in the ICU *Crit Care Med*2014;46**(6)**:1 doi 10.1097/CCM.0000000000003067.

19 Lalfond S, Charlesworth. A Year of plenty? (2017) The Health Foundation. London.

20 Hewitt C, Lankshear A, Kazanjn A *et al.* Health Service Workforce and Health Outcomes: A Scoping Study. Report for the National Co-ordinating Centre for NHS Service Delivery and Organisation. https://www.journalslibrary.nihr.ac.uk/programmes/hsdr/081319050/#/documentation accessed May 2019

21 Huang D, Clermont G, Sexton K *et al.* Perceptions of safety culture vary across the intensive care units of a single institution. *Crit Care Med*2007; 35**1:**165-176 doi10.1097/01.CCM.0000251505.76026.CF.

22 D'Lima D, Murray E, Brett S. Perceptions of Risk and Safety in the ICU: A Qualitative Study of Cognitive Processes Relating to Staffing *Crit Care Med*2018*;*46**1**:60-70. doi10.1097/CCM.0000000000002773.

23 Wolf L, Perhats C, Delao A *et al*. On the Threshold of Safety: A Qualitative Exploration of Nurses' Perceptions of Factors Involved in Safe Staffing Levels in Emergency Departments. *Journal of Emergency Nursing*2017;43**2:**150-157 doi 10.1016/j.jen.2016.09.003.

24 Vandevala T, Pavey L, Chelidoni O *et al.* Psychological rumination and recovery from work in intensive care professionals: Associations with stress, burnout, depression and health. *Journal of Intensive Care*2017;5**16** doi 10.1186/s40560-017-0209-0.

For peer review only

25 Seaman J, Cohen T, White. D. Reducing the stress on clinicians working in the ICU *JAMA* 2019; 320**19**:1981-1982 doi10.1001/jama.2018.14285.

26 Halter M, Boiko O, Pelone f *et al.* The determinants and consequences of adult nursing staff turnover a systematic review of systematic reviews *Health Services Research.* 17**1**:824 doi 10.1186/s12913-017-2707-0.

27 National Institute for Health Research Themed Review. Staffing on Wards. Making decisions about healthcare staffing improving effectiveness and supporting staff to care well. *NIHR Dissemination Centre* 2019 doi 10.3310/themedreview-03553La

28 Critical Care Minimum Dataset. Available from: http://www.datadictionary.nhs.uk/data_dictionary/messages/supporting_data_sets/data_sets/critical_care_minimu m_data_set_fr.asp (accessed December2018)

29 NHS Improvements *Care hours per patient day (CHPPD): guidance for acute and acute specialist trusts (*2018) NHS Improvements https://improvement.nhs.uk/documents/3177/CHPPD_guidance_-_acute.pdf (accessed April 2019).

30 The Shelford Group. Safer nursing care tool implementation resource pack. The Shelford Group 2014.

31 The Health Foundation Measuring safety culture. The Health Foundation 2011.

32 Olds D, Aiken L, Cimiotti J *et al.* Association of nurse work environment and safety climate on patient mortality: A cross-sectional study. *Int J Nurs Stud*2017;**(74):**155-161 doi 10.1016/j.ijnurstu.2017.06.004

33 Wong A, Olusanya O. Burnout and resilience in anaesthesia and intensive care medicine. *BJA Education*2017;17**10:** 334-340 doi 10.1093/bjaed/mkx020

34 Ball J, Barker H, Griffiths P*et al.* Implementation, Impact and Costs of Policies for Safe Staffing in Acute NHS Trusts 2019 doi:10.5258/SOTON/P0012

35 Saville C, Griffiths P, Ball J *et al.* How many nurses do we need? A review and discussion of operational research techniques applied to nurse staffing. *Int J Nurs Stud*2019; **97:**7-13. doi10.1016/j.ijnurstu.2019.04.015

36 Maben J, Griffiths P, Penfold C *et al*. One size fits all? Mixed methods evaluation of the impact of 100% single-room accommodation on staff and patient experience, safety and costs *BMJ Qual Saf* 2016;**2**:241-256 doi:10.1136/bmjqs-2015-004265

37 Maben J, Griffiths P, Penfold C, *et al*. Evaluating a major innovation in hospital design: workforce implications and impact on patient and staff experiences of all single room hospital accommodation. *Health Serv Deliv Res*.2015;**3**:304. doi 10.3310/hsdr03030

38 El Khamali R, Mouaci A, Valera S, *et al;* for the SISTRESSREA Study Group. Effects of a multimodal program including simulation on job strain among nurses working in intensive care units: a randomized clinical trial. *JAMA2018;*.**320**:1988-1997doi:10.1001/jama.2018.14284

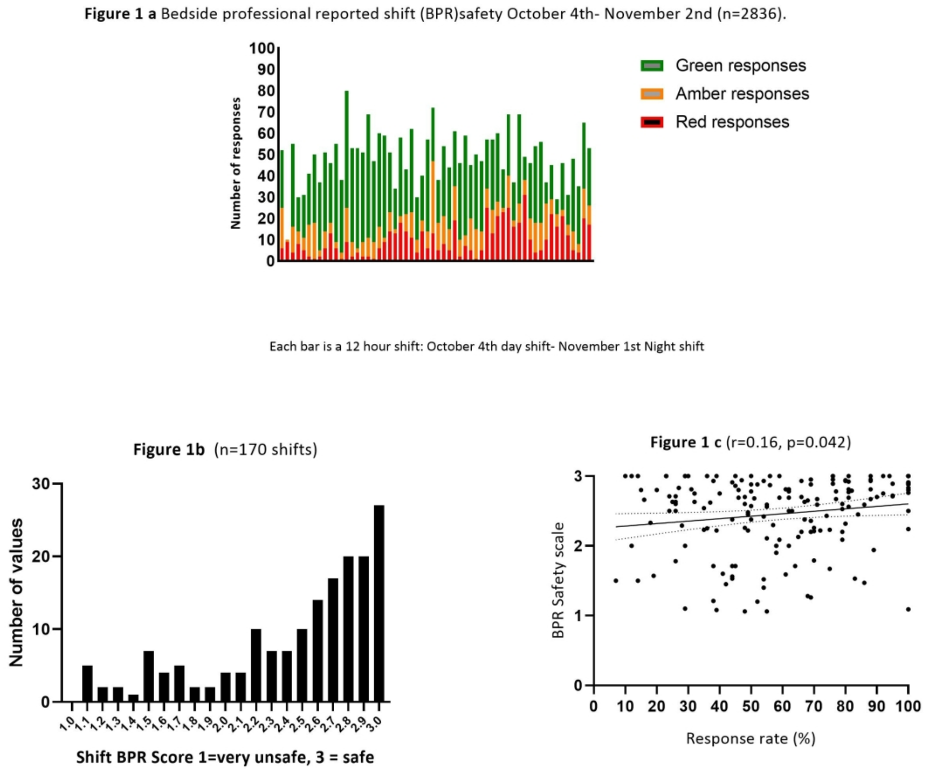


Figure 1a Bedside professional reported (BPR) safety October 4th-November 2nd (n=2836).
Figure 1b Distribution of bedside professional reported (BPR) safety - shift scores (n=170/ 174 shifts).
Figure 1 c Relationship between bedside professional reported (BPR) safety and response rates (n=170/ 174 shifts).

215x178mm (300 x 300 DPI)

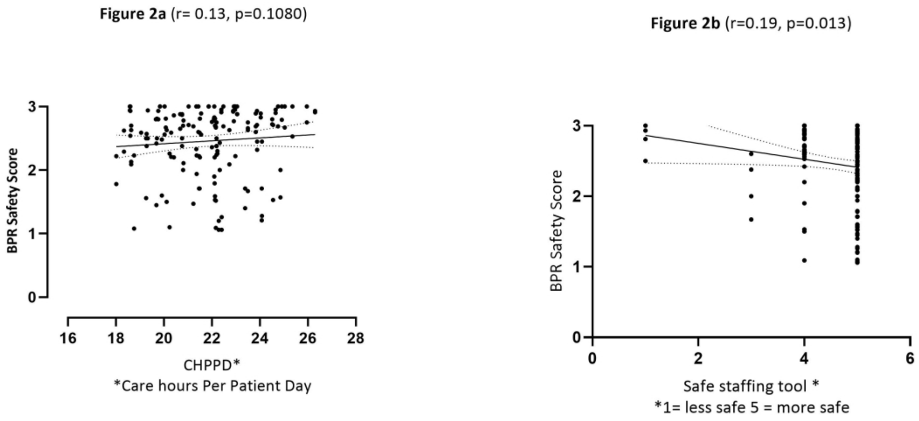


Figure 2a Relationship between bedside professional reported (BPR) shift safety and care hours per patient day (CHPPD) (n=170/ 174 shifts)
Figure 2b Relationship between bedside professional reported (BPR) shift safety (BPR) and safe staffing "score" 1=staff overutilization red, 5 = green staff underutilization (n=170/ 174 shifts)

252x119mm (300 x 300 DPI)

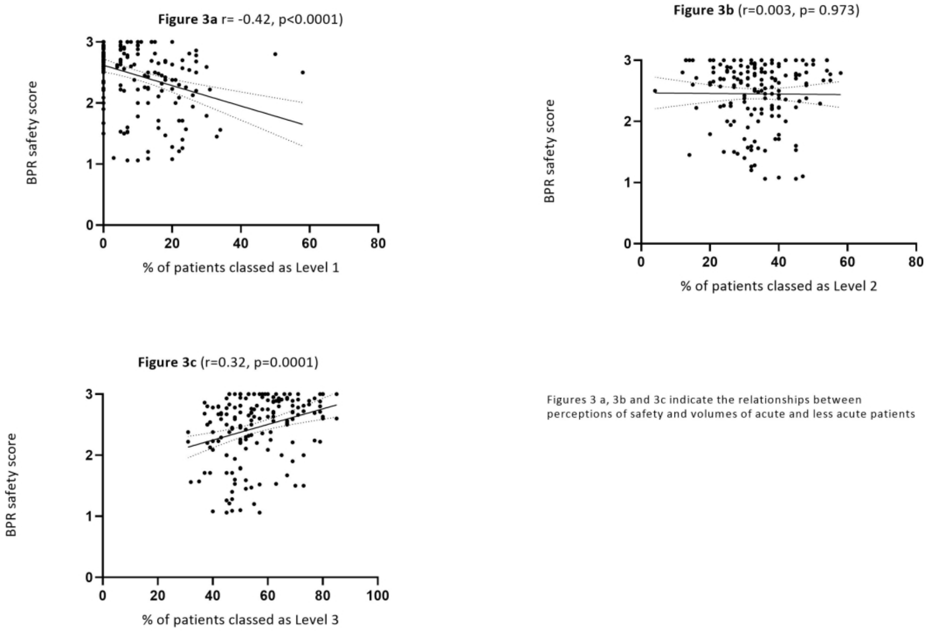


Figure 3 a, b & c Relationship between bedside professional reported (BPR) shift safety and volumes of acute and less acute patients Level 1, Level 2, Level 3 (n=170/ 174 shifts)

251x171mm (300 x 300 DPI)

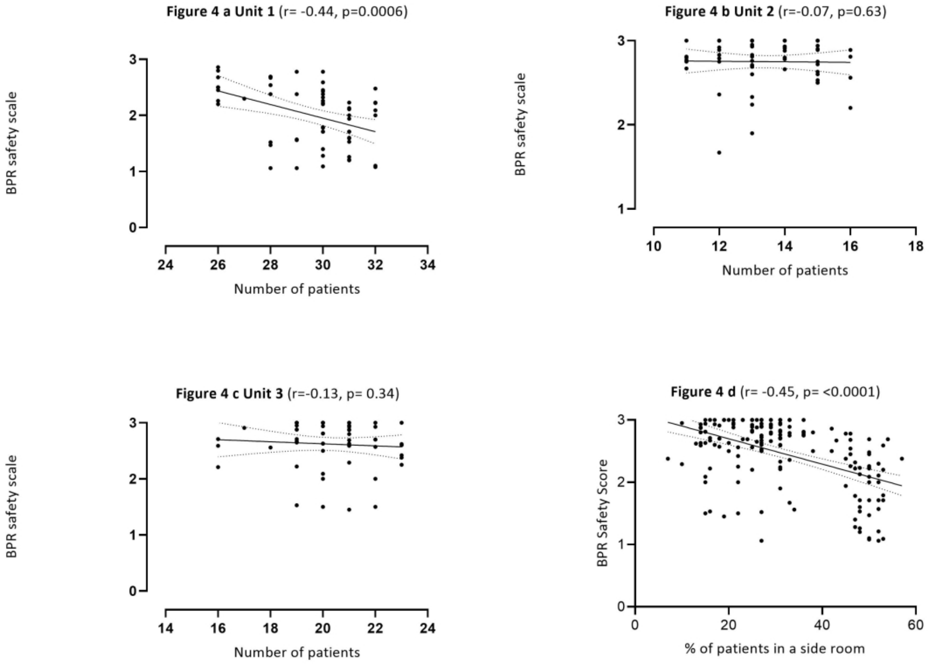


Figure 4 a, b & c Relationships between bedside professional reported (BPR) shift safety and number of patients in unit 1 (n=58), 2(n=56) and 3(n=56) Figure 4d Relationship between bedside professional reported perceptions of shift safety (BPR) and the percentage of patients in a side room (n=170/ 174 shifts).

252x180mm (300 x 300 DPI)

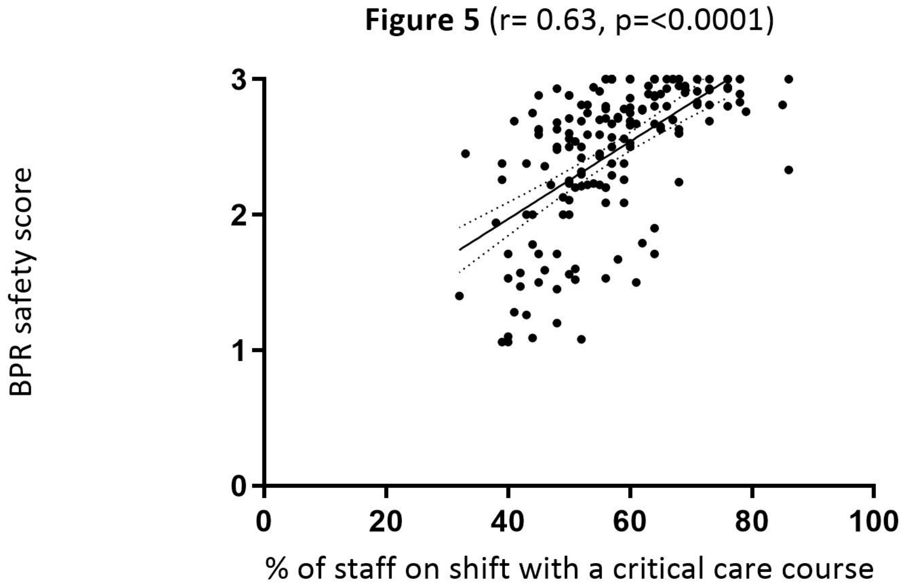


Figure 5 Relationship between bedside professional reported (BPR) shift safety and the percentage of staff on a shift with a critical care course (n=170/ 174 shifts).

124x80mm (300 x 300 DPI)

**SUPPLEMENTARY MATERIALS**

**Supplementary File**

**Supplementary Table 1 Department of Health 2009 Levels of Care classification**

| | |
|---|---|
| Level 0 | Patients whose needs can be met through normal ward care in an acute hospital |
| Level 1 | Patients at risk of their condition deteriorating, or those recently relocated from higher levels of care, whose needs can be met on an acute ward with additional advice and support from the Critical Care team |
| Level 2 | Patients requiring more detailed observation or intervention including support for a single failing organ system or post-operative care and those 'stepping down' from higher levels of care |
| Level 3 | Patients requiring advanced respiratory support alone, or basic respiratory support together with support of at least two organ systems. This level includes all complex patients requiring support for multi-organ failure. |

**Supplementary Table 2 Characteristics of ICU sites during data collection**

| Site | Number of available beds | Number of side rooms | Core Specialities |
|---|---|---|---|
| Site 1 | 32 | 16 | Major Trauma Centre, Vascular, General |
| Site 2 | 16 | 4 | Heart Attack Centre, Hepatobiliary, Renal and transplant, Haematology/Oncology |
| Site 3 | 24 | 5 | Stroke centre, Neurological, Neurosurgical, Head and Neck surgery, General. |

For peer review only

**Supplementary Table 3a Safer Nursing Care Tool**

| Level of Care | Descriptor |
|---|---|
| Level 0<br>Patient requires hospitalisation Needs met by provision of normal ward carers. | **Care requirements may include the following**<br>**• Elective medical or surgical admission**<br>**• May have underlying medical condition requiring on-going treatment**<br>**• Patients awaiting discharge**<br>**• Post-operative/post-procedure care - observations recorded half hourly initially then 4-hourly**<br>**• Regular observations 2 - 4 hourly**<br>**• Early Warning Score is within normal threshold.**<br>**• ECG monitoring**<br>**• Fluid management**<br>**• Oxygen therapy less than 35%**<br>**• Patient controlled analgesia**<br>**• Nerve block**<br>**• Single chest drain**<br>**• Confused patients not at risk**<br>**• Patient requires assistance with some activities of daily living, requires the assistance of one person to mobilise, or experiences occasional incontinence** |
| Level 1a<br>Acutely ill patients requiring intervention or those who are UNSTABLE with a GREATER POTENTIAL to deteriorate | **Care requirements may include the following**<br>**• Increased level of observations and therapeutic interventions**<br>**• Early Warning Score - trigger point reached and requiring escalation.**<br>**• Post-operative care following complex surgery**<br>**• Emergency admissions requiring immediate therapeutic intervention.**<br>**• Instability requiring continual observation/invasive monitoring**<br>**• Oxygen therapy greater than 35% +/- chest physiotherapy 2-6 hourly**<br>**• Arterial blood gas analysis - intermittent**<br>**• Post 24 hours following insertion of tracheostomy, central lines,epidural or multiple chest or extra ventricular drains**<br>**• Severe infection or sepsis** |
| Level 1b<br>Patients who are in a STABLE condition but are dependant on nursing care to meet most or all of the activities of daily living | **Care requirements may include the following**<br>**• Complex wound management requiring more than one nurseor takes more than one hour to complete.**<br>**• VAC therapy where ward-based nurses undertake the treatment**<br>**• Patients with Spinal Instability /Spinal Cord Injury**<br>**• Mobility or repositioning difficulties requiring the assistance of two people**<br>**• Complex Intravenous Drug Regimes - (including those requiring prolonged preparatory /administration/post-administration care)**<br>**• Patient and/or carers requiring enhanced psychological support owing to poor disease prognosis or clinical outcome**<br>**• Patients on End of Life Care Pathway** |

For peer review only

| | |
|---|---|
| | • Confused patients who are at risk or requiring constant supervision<br>• Requires assistance with most or all activities of daily living<br>• Potential for self-harm and requires constant observation<br>• Facilitating a complex discharge where this is the responsibility of the ward-based nurse |
| Level 2<br>May be managed within clearly identified, designated beds, resources with the required expertise and staffing level OR may require transfer to a dedicated Level 2 facility /unit | **Deteriorating/ compromised single organ system**<br>• **Post operative optimisation (pre-op invasive monitoring)/extended post-op care.**<br>• **Patients requiring non-invasive ventilation/respiratory support;**<br>**CPAP/BiPAP in acute respiratory failure**<br>• **First 24 hours following tracheostomy insertion**<br>• **Requires a range of therapeutic interventions including:**<br>• **Greater than 50% oxygen continuously**<br>• **Continuous cardiac monitoring and invasive pressure monitoring**<br>• **Drug Infusions requiring more intensive monitoring e.g. vasoactive drugs (amiodarone, inotropes, gtn) or potassium, magnesium**<br>• **Pain management - intrathecal analgesia**<br>• **CNS depression of airway and protective reflexes**<br>• **Invasive neurological monitoring** |
| Level 3<br>Patients needing advanced respiratory support and/or therapeutic support of multiple organs. | **Monitoring and supportive therapy for compromised/ collapse of two or more organ/ systems**<br>• **Respiratory or CNS depression/ compromise requires mechanical/invasive ventilation**<br>• **Invasive monitoring, vasoactive drugs, treatment of hypovolaemia/haemorrhage/ sepsis or neuro protection** |
| | |

For peer review only

**Supplementary Table 3b**

| SafeCare Allocate Colour | SafeCare Allocate Calculation | Category for analysis |
|---|---|---|
| Green | 90% (underutilisation ) | 5 |
| None given | 90.1%-105% | 4 |
| Amber | 105 % (overutilisation) | 3 |
| None Given | 105.1%-110% | 2 |
| Red | >110% (overutilisation) | 1 |

**Supplementary Table 4 Summary of Responses**

| | Total | | Site 1 | | Site 2 | | Site 3 | |
|---|---|---|---|---|---|---|---|---|
| | Total | % | Total | % | Total | % | Total | % |
| Green | 1731 | 61% | 495 | 39% | 685 | 83% | 551 | 74% |
| Amber | 523 | 18% | 304 | 24% | 101 | 12% | 118 | 16% |
| Red | 582 | 21% | 461 | 37% | 44 | 5% | 77 | 10% |
| Total responses | 2836 | | 1260 | | 830 | | 746 | |
| Response rate | 57.7% | | 56.4% | | | 70.8% | | 46.0% |

For peer review only

**Supplementary Table 5** Analysis of relationships between BPR and staffing, patient and work environment characteristics using mean, median and weighted mean BPR score.

| Variable tested | BPR Score Mean* | | BPR Score Median** | | BPR Score Mean weighted *** | |
|---|---|---|---|---|---|---|
| | r value | p value | r value | p value | r value | p value |
| **CHPPD** | 0.13 | 0.108 | 0.01 | 0.885 | -0.11 | 0.151 |
| **Safe care Allocate Score** | -0.19 | 0.013 | -0.17 | 0.032 | 0.17 | 0.032 |
| **% of level 1 patients** | -0.42 | <0.0001 | -0.34 | <0.0001 | 0.41 | <0.0001 |
| **% of level 2 patients** | 0.003 | 0.973 | 0.02 | 0.78 | -0.0004 | 0.996 |
| **% of level 3 patients** | 0.32 | <0.0001 | 0.22 | 0.004 | -0.32 | <0.0001 |
| **Number of patients** | -0.6 | <0.0001 | -0.54 | <0.0001 | 0.59 | <0.0001 |
| **% of patients in side rooms** | -0.45 | <0.0001 | -0.39 | <0.0001 | 0.44 | <0.0001 |
| **% of staff with a CCC** | 0.63 | <0.0001 | 0.55 | <0.0001 | 0.61 | <0.0001 |

***each green response scores 3, each amber response scores 2, each red response scores 1, total score divided by the number of responses= BPR shift score. (3=safe 1 = very unsafe)**
****each green scores 3, each amber scores 2, each red scores 1, median score used= BPR shift score (3 =safe 1 =very unsafe)**
***** each green response scores 1, each amber scores 3, each green response scores 5, total score divided by the number of responses= BPR shift score (1= safe 5 = very unsafe)**

For peer review only

**Supplementary Figure 1**

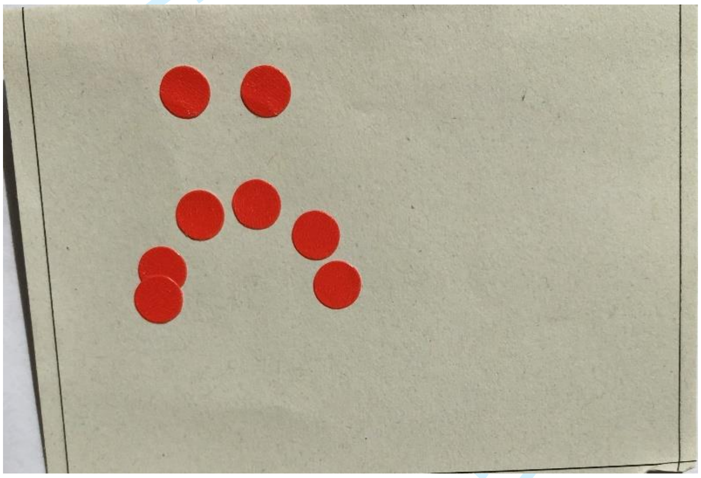

Image 1

For peer review only

For peer review only
